# Is autophagy always a death sentence? A case study of highly selective cytoplasmic degradation during phloemogenesis

**DOI:** 10.1093/aob/mcae195

**Published:** 2024-11-05

**Authors:** Kornel M Michalak, Natalia Wojciechowska, Karolina Kułak, Julia Minicka, Andrzej M Jagodziński, Agnieszka Bagniewska-Zadworna

**Affiliations:** Department of General Botany, Institute of Experimental Biology, Faculty of Biology, Adam Mickiewicz University in Poznań, Uniwersytetu Poznańskiego 6, Poznań 61-614, Poland; Department of General Botany, Institute of Experimental Biology, Faculty of Biology, Adam Mickiewicz University in Poznań, Uniwersytetu Poznańskiego 6, Poznań 61-614, Poland; Department of General Botany, Institute of Experimental Biology, Faculty of Biology, Adam Mickiewicz University in Poznań, Uniwersytetu Poznańskiego 6, Poznań 61-614, Poland; Department of Virology and Bacteriology, Institute of Plant Protection in Poznań, Węgorka 20, Poznań 60-318, Poland; Department of Ecology, Institute of Dendrology, Polish Academy of Sciences, Parkowa 5, Kórnik 62-035, Poland; Department of General Botany, Institute of Experimental Biology, Faculty of Biology, Adam Mickiewicz University in Poznań, Uniwersytetu Poznańskiego 6, Poznań 61-614, Poland

**Keywords:** Phloem development, sieve elements, selective autophagy, plastolysomes, enucleation, plant roots, *Populus trichocarpa*

## Abstract

**Background and Aims:**

The transformation of sieve elements from meristematic cells, equipped with a full complement of organelles, to specialized transport tubes devoid of a nucleus has long been enigmatic. We hypothesized a strong involvement of various degradation pathways, particularly macroautophagy in this context, emphasizing the importance of autophagic selectivity in the remaining viability of these cells.

**Methods:**

Experiments were performed on pioneer roots of *Populus trichocarpa* cultivated in rhizotrons under field conditions. Through anatomical, ultrastructural and molecular analyses, we delineate the stages of phloemogenesis and the concurrent alterations in the cytoplasmic composition of SEs.

**Key Results:**

Notably, we observed not only macroautophagic structures, but also the formation of autophagic plastids, the selective degradation of specific organelles, vacuole disruption and the release of vacuolar contents. These events initially lead to localized reductions in cytoplasm density, but the organelle-rich cytoplasmic phase is safeguarded from extensive damage by a membrane system derived from the endoplasmic reticulum. The sieve element ultimately develops into a conduit containing electron-translucent cytoplasm. Eventually, the mature sieve element is a tube filled only by translucent cytoplasm, with sparse organelles tethered to the cell wall.

**Conclusions:**

Although the activation of programmed cell death pathways was postulated, the persistence of sieve elements indicates that protoplast depletion is meticulously regulated by hitherto unidentified mechanisms. This research elucidates the sequential processes occurring in these cells during phloemogenesis and unveils novel insights into the mechanisms of selective autophagy.

## INTRODUCTION

The plant vasculature comprises a complex network of various cell types with a shared origin, tasked with distributing resources throughout the plant. The xylem primarily transports water and minerals, while the phloem is responsible for assimilate allocation (Scarpella and Meijer, 2004). The development of conducting cells within these heterogeneous tissues involves both constructive and destructive processes (Wojciechowska *et al.*, 2021). Xylem tracheary elements (TEs), for instance, undergo programmed cell death (PCD), utilizing autophagic and autolytic pathways (Kwon *et al.*, 2010*a*; Bagniewska-Zadworna *et al.*, 2012; Wojciechowska *et al.*, 2019). This process is characterized by the gradual removal of organelles via autophagy, and the flux of cellular component elimination and enzymatic recycling (van Doorn and Woltering, 2010; Minina *et al.*, 2014), accompanied by vesicle accumulation and vacuole disintegration, leading to cytosolic acidification and ultimate lysis (Ameisen, 2002; Kuriyama and Fukuda, 2002; Lam, 2004; Della Mea *et al.*, 2007; Daneva *et al.*, 2016). Autophagy thus plays a dual role: it is essential for both cell survival and cell death. In contrast, phloem sieve elements (SEs), which are living cells devoid of most organelles at maturity, present a unique case (Aloni, 1987). These elongated tubes are adapted for long-distance transport, containing only minimal cytoplasm and lacking a nucleus, Golgi apparatus, vacuoles and other organelles. The peripheral regions of SEs house a few remaining plastids or mitochondria, vesicles of unidentified origin, and fragmented membranes (Eleftheriou, 1986; Evert, 2006; Heo *et al.*, 2017). Sieve elements are further characterized by tissue-specific features such as extensive endoplasmic reticulum (ER) aggregates, phloem-specific P-proteins of various types (Cronshaw and Sabnis, 1990), and sieve plates containing callose deposits and pores connected by transcellular cytoplasmic strands (Johnson *et al.*, 1976). Originating from meristematic cells rich in dense cytoplasm with a centrally located nucleus and abundant organelles, SE development relies heavily on degradation processes that reduce organelle numbers and do not culminate in cell death. This phenomenon was termed ‘programmed cell semi-death’ (van Bel, 2003). The mechanisms by which SEs maintain viability, the regulation of selective autophagy (Evert, 1977), selective autolysis (Yin and Fan, 2008) and the cessation of these processes to prevent cell death remain unclear. The vacuole is presumed to play a crucial role in this context, with increased vacuolization, vacuole enlargement and tonoplast disintegration alongside nuclear structure alterations. These observations corresponded to nucleus degradation confirmed by the TUNEL (terminal deoxynucleotidyl transferase-mediated dUTP nick end-labelling) assay (Wang *et al.*, 2008). In developing SEs of *Triticum aestivum*, protease activity and protoplast acidification coincide with vacuole volume expansion (Yang *et al.*, 2015). Conversely, in *Arabidopsis thaliana* SEs, multiple small lytic vacuoles are observed rather than a single large central vacuole. Notably, nuclear degradation occurs only after other organelle rearrangement and the degradation of cytoplasm (Furuta *et al.*, 2014). Autophagic events have been thoroughly documented in the development of TEs in the roots and stems of *Populus trichocarpa*, with the presence of autophagy-related ATG8 protein indicating autophagy’s role in xylogenesis and in the phloem (Wojciechowska *et al.*, 2019). However, the involvement of various autophagic mechanisms or PCD-like processes in phloemogenesis, as compared with xylogenesis, lacks comprehensive evidence.

The detection of ATG8 in the phloem suggests that macroautophagy may also contribute to and drive SE development. Recent findings reveal that autophagy’s role in the development of TEs, which are dead at maturity, and SEs, which remain alive, is universal for the plant kingdom, as confirmed across various taxonomic groups [ferns, gymnosperms, angiosperms (monocotyledonous, dicotyledonous, both herbaceous and woody)] (Michalak *et al.*, 2024). Structurally, autophagy typically initiates with the sequestration of a portion of the cytoplasm by a double-membrane structure known as the phagophore. This structure expands and seals to form an autophagosome, which is then delivered to the vacuole, where its outer membrane fuses with the tonoplast, releasing the autophagic body for digestion within the vacuole. Such phenomena, however, have yet to be described in differentiating phloem cells. In contrast, all types of autophagy mediate cytoplasmic degradation during xylogenesis, resulting in TEs that become dead, hollow, and specialized for substance transport (Kwon *et al.*, 2010*b*; Bagniewska-Zadworna *et al.*, 2012, 2014; Escamez *et al.*, 2016; Wojciechowska *et al.*, 2019). The involvement of similar mechanisms in the differentiation of other conducting cells remains unclear. This study hypothesizes that phloemogenesis involves a unique differentiation programme, potentially engaging mechanisms akin to the PCD observed in xylogenesis, yet not resulting in cell death. The sequence and timing of events leading to the progressive degradation of protoplast components, which undergo dramatic changes during SE development, are yet to be fully elucidated. The mechanisms ensuring SE survival are also unknown. This research introduces a novel concept positing that macroautophagy plays a significant role in the degradation processes during phloem conductive cell development. It aims to comprehensively identify selective autophagy symptoms and, importantly, uncover new mechanisms of cytoplasmic removal and the fate of individual organelles during SE development in *P*. *trichocarpa* root.

## MATERIALS AND METHODS

### Plant material and growth conditions

**Fig. 1.**
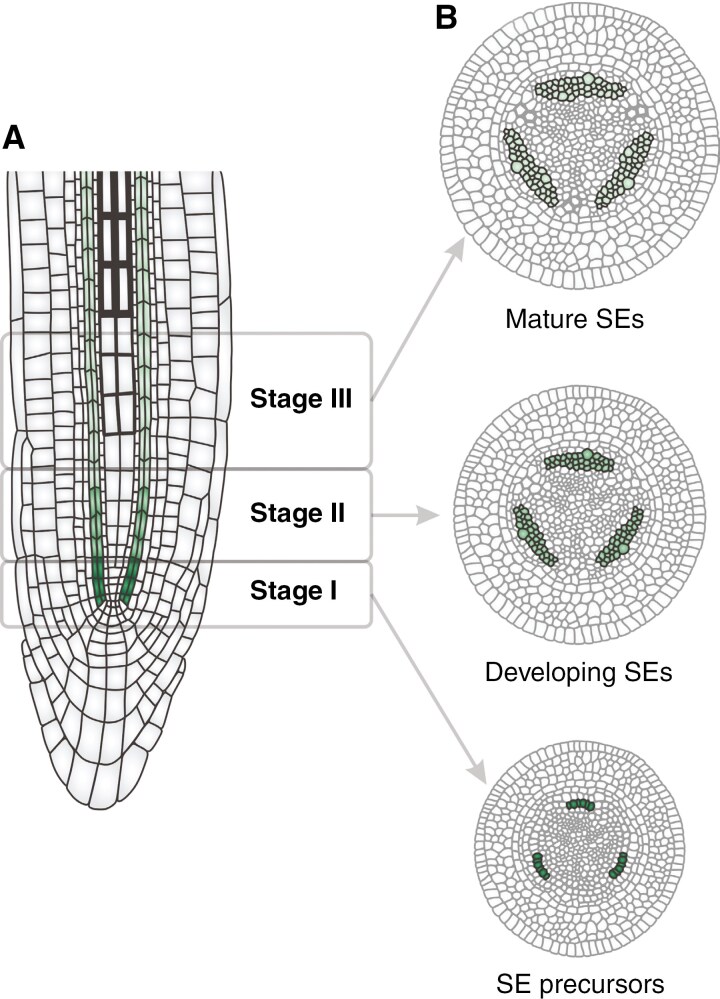
Illustrative overview of phloemogenesis stages in the pioneer root of *P. trichocarpa*, depicted in selected root fragments according to the differentiation stages of SEs. (A) Tangential section. (B) Transverse sections. The phloem is represented in a gradient of green shades, from dark green for SE precursors to the lightest green for mature SEs; illustration not drawn to scale.

Experiments were conducted on pioneer roots of black cottonwood (*Populus trichocarpa*), with plants propagated from seeds sourced from seed banks. Seedlings were initially grown in a Conviron GR96 plant growth chamber, under a 16-h day/8-h night photoperiod, at temperature of 18 °C during the day and 14 °C at night. Subsequently, the plants were transferred to an experimental field site located at the Institute of Dendrology, Polish Academy of Sciences in Kórnik, Poland (52°14ʹ40ʹʹN, 17°06ʹ27ʹʹE). Here, plants were cultivated in rhizotrons positioned vertically within an underground chamber, set up in a semi-open, foil tunnel greenhouse according to the protocol described in Wojciechowska *et al.* (2018). Pioneer roots were harvested and segmented into small fragments corresponding to the developmental stages (Fig. 1) of the primary structure as outlined in Bagniewska-Zadworna *et al.* (2014) and the conserved pattern of phloem differentiation of eudicots (Ostermeyer *et al.*, 2022).

### Light microscopy

Root fragments, corresponding to each developmental stage of SEs, were immediately fixed in a fresh mixture containing 2 % (v/v) glutaraldehyde and 2 % (v/v) formaldehyde diluted in 0.1 m cacodylic buffer (pH 7.3; Polysciences), followed by overnight incubation at 4 °C. After fixation, the samples were washed three times with a 0.05 m cacodylate buffer, then dehydrated in a graded series of ethanol (10–100 %, v/v). For the preparation of thin cross-sections, the roots were embedded in Technovit 7100 resin (Heraeus Kulzer, Wehrheim, Germany), following a series of incubations in ethanol:resin mixtures with ratios of 3:1, 1:1 and 1:3, and finally in pure resin with the addition of a hardener (Heraeus Kulzer). After 48 h of polymerization at room temperature, sections were cut at a thickness of 8 μm using a Leica RM2265 fully automated rotary microtome (Leica-Reichert, Bensheim, Germany) and mounted on glass slides. The sections were then stained with 0.5 % (m/v) toluidine blue and examined under a light microscope (Axioscope A1, Carl Zeiss, Jena, Germany).

### Transmission electron microscopy

The fixation protocol for transmission electron microscopy (TEM) analysis was identical to that described for light microscopy (LM), with additional steps in accordance with the methods for the phloem (Hunziker and Schulz, 2019), with slight modifications optimized for pioneer roots of *P. trichocarpa*. Samples were transferred into post-fixation solution of 1 % osmium tetroxide in 0.1 m cacodylic buffer (pH 7.3; Polysciences) for 2 h and counterstained with 2 % uranyl acetate in 5 % ethanol for 1 h at room temperature in darkness. Following dehydration in a graded ethanol series (10–100 %, v/v), the samples were infiltrated with a series of ethanol:acetone mixtures (3:1, 1:1, 1:3), culminating in 100 % acetone, then embedded in Spurr’s low-viscosity resin (Polysciences) by incubation in a series of acetone:resin mixture with ratio 1:1 and two rinses in pure resin. Polymerization was carried out at 60 °C for 72 h. Ultrathin sections were prepared using an ultramicrotome (EM UC6, Leica-Reichert) with a diamond knife, at a thickness of 0.1 μm, and placed on formvar-coated copper grids. The sections were stained with 2 % uranyl acetate and 2 % lead citrate, and examined with an HT7700 transmission electron microscope (Hitachi, Tokyo, Japan) operating at an accelerating voltage of 100 kV. The analysis proceeded from mature SEs towards the root tip to ensure certainty of the cell type. For comprehensive ultrastructural analysis, at least five samples from each developmental stage were examined. Due to their elongated structure, the same sieve tube was cross-sectioned at different heights to ensure no changes occurring throughout the SE were missed. The traits that were repeated in at least ten different sections or section series from different samples and plants were considered as confirmed.

### Gene identification

Gene sequences were identified within the *P. trichocarpa* genome by comparative analysis with peptide sequences encoded by known genes of *A. thaliana*. Specifically, the sequences of genes encoding ATG8 proteins in *P. trichocarpa* were determined based on a previous phylogenetic overview (Kellner *et al.*, 2017). A phylogenetic tree was constructed using MEGA 11 software (Molecular Evolutionary Genetics Analysis ver. 11.0.11 by Tamura *et al.* (2021)), applying the neighbour-joining statistical method and the bootstrap method for phylogeny testing.

### RT–qPCR analysis of gene expression

RNA isolation was conducted using a Ribospin Plant kit (GeneAll Biotechnology Co., Ltd, Seoul, South Korea), adhering to the manufacturer’s protocol. cDNA synthesis was performed with a High-Capacity cDNA Reverse Transcription Kit (Applied Biosystems, Thermo Fisher Scientific Inc., Waltham, MA, USA), following the supplied protocol. RT–qPCR was executed using a SYBR Green Master Mix kit (Applied Biosystems) in a CFX96 Touch Real-Time PCR Detection System (Bio-Rad, Hercules, CA, USA). The amplification programme consisted of an initial denaturation by hot start at 95 °C for 10 min followed by 40 cycles of a two-step program (denaturation at 95 °C for 15 s and annealing/extension at 60 °C for 1 min). *ACTIN* and *UBQ* (ubiquitin) were selected as housekeeping genes for the normalization of expression values, chosen for their minimal sample-to-sample variation and stable expression across all sample types and time points. The sequences of the gene-specific primer pairs used in the RT–qPCR analyses are listed in Supplementary Data Table S1. Genes exhibiting changes in expression within the developmental stages were selected for further analysis. Data analyses were performed in accordance with the method previously described in Bagniewska-Zadworna and Stelmasik (2015). All gene expression analyses utilized three technical replicates for each of the three biological replicates of each experimental variant. Statistical analyses (Supplementary Data Fig. S1) were conducted using Statistica 10 software (StatSoft Poland Inc., Tulsa, OH, USA).

### Immunohistochemistry

For immunohistochemical detection, 40-μm-thick root slices were prepared using a Leica VT 1200S vibratome (Leica Biosystems) as reported by Singh *et al.* (2022). Sections were fixed in 4 % (v/v) formaldehyde and 0.5 % (v/v) glutaraldehyde in 0.1 m phosphate-buffered saline (PBS) buffer (pH 6.8; Polysciences) for 2 h, then rinsed in 0.05 m PBS. For membrane permeabilization, the material was incubated for 15 min in 0.1 % Triton (Polysciences). The material was treated with antibodies according to the procedure described by Wojciechowska *et al.* (2019). A primary ATG8 rabbit antibody (cat. no. AS14 2769; Agrisera, Sweden) was used, and the tyramide signal amplification (TSA) technique was employed for its high sensitivity, followed by an incubation in a poly-HRP-conjugated secondary antibody (Thermo Fisher Scientific Inc., USA, attached to the TSA Super Boost kit) as reported by Wojciechowska *et al.* (2018). Immunohistochemical detection of SE cell walls was performed for unquestionable localization of these cells within phloem. Highly specific LM26 rat antibody (recognizing a 1,6-galactosyl substitution in 1,4-β-d-galactans; cat. no. AS22 4808; Agrisera) was used, followed by an incubation with Goat Anti-Rat IgG (H + L) secondary antibody (Alexa Fluor^®^ 546 Conjugate, cat. no. A-11081; Thermo Fisher Scientific Inc.) according to the procedure described by Marzec-Schmidt *et al.* (2019). Imaging of ATG8 localization was performed with a Leica TCS SP5 confocal microscope (Leica Biosystems, Nussloch, Germany), utilizing an argon laser at wavelength 488 nm. Results for LM26 were recorded using a Leica Stellaris DMi8 confocal microscope (Leica Biosystems) utilizing a white light laser (WLL) emitting light at 546 nm. In the control reactions, incubations with the primary antibodies were omitted (Supplementary Data Figs S2 and S3).

### Immunocytochemistry

Root fragments measuring 0.5 cm were ﬁxed in 0.5 % glutaraldehyde and 4 % formaldehyde in 0.1 m PBS (pH 6.8; Polysciences) for 12 h at 4 °C. The osmium tetroxide and uranyl acetate treatment steps were omitted to maintain necessary reaction contrast. Following fixation, the samples were rinsed in 0.05 m PBS, dehydrated in a graded ethanol series and embedded in LR White resin (Sigma). Polymerization was carried out at 4 °C with UV light for 72 h in a PELCO^®^ UVC3 Cryo Chamber (USA). Ultrathin sections (60 nm) were cut using a Leica EM UC6 ultramicrotome (Leica Biosystems) with a diamond knife, and collected on formvar-coated nickel grids. The material was blocked in 1 % acetylated bovine serum albumin (acBSA) in PBS for 15 min at room temperature. After blocking, sections were incubated with a primary rabbit antibody against ATG8 protein, diluted 1:50 in 0.05 % acBSA. Following washing in PBS, the sections were incubated with 10 nm gold-labelled goat anti-rabbit secondary antibody (cat. no. G7402; Sigma) diluted 1:20 in 0.05 % acBSA in PBS at 37 °C for 2 h. Sections were then stained with 2 % uranyl acetate for 10 min and 2 % lead citrate for 10 min, and examined with an HT7700 transmission electron microscope (Hitachi) operating at an accelerating voltage of 100 kV. For cytological studies, ten root segments were embedded, and at least three grids for each segment were examined under a transmission electron microscope. In the control reactions, incubations with the primary antibodies were omitted (Supplementary Data Fig. S4).

### Terminal deoxynucleotidyl transferase-mediated dUTP nick end-labelling assay

The TUNEL assay was employed to detect cells undergoing nuclear degradation, as indicated by green fluorescence marking controlled DNA fragmentation sites. Samples were fixed in a mixture of 2 % (v/v) glutaraldehyde and 2 % (v/v) formaldehyde (pH 6.8; Polysciences) for 12 h at 4 °C. Following fixation, the samples were dehydrated in a graded ethanol series and subsequently embedded in Paraplast X-TRA^®^ (Sigma–Aldrich). Cross-sections of 15 μm thickness were prepared using a Leica RM2265 fully automated rotary microtome. Paraplast was removed by incubation in xylene (Sigma) and an ethanol series. The Click-iT™ Plus TUNEL Assay Kits for In Situ Apoptosis Detection (C10617; Applied Biosystems) were utilized according to the manufacturer’s instructions, with slight modifications. TUNEL reactions were conducted for 40 min in darkness at 37 °C. DNA was stained using DAPI (D1306, Applied Biosystems). A minimum of 15 root segments were analysed. Positive controls were established by inducing DNA strand breaks using DNase Reaction Buffer (Qiagen), whereas negative controls were prepared by omitting the TdT reaction mixture during incubation (Supplementary Data Fig. S5). Imaging was performed with a Leica TCS SP5 confocal microscope (Leica Biosystems, Nussloch, Germany) with an argon laser emitting light at 488 nm for results of the TUNEL assay and a 405-nm diode laser for results of DAPI staining.

## RESULTS

### Determination of phloemogenesis stages

**Fig. 2.**
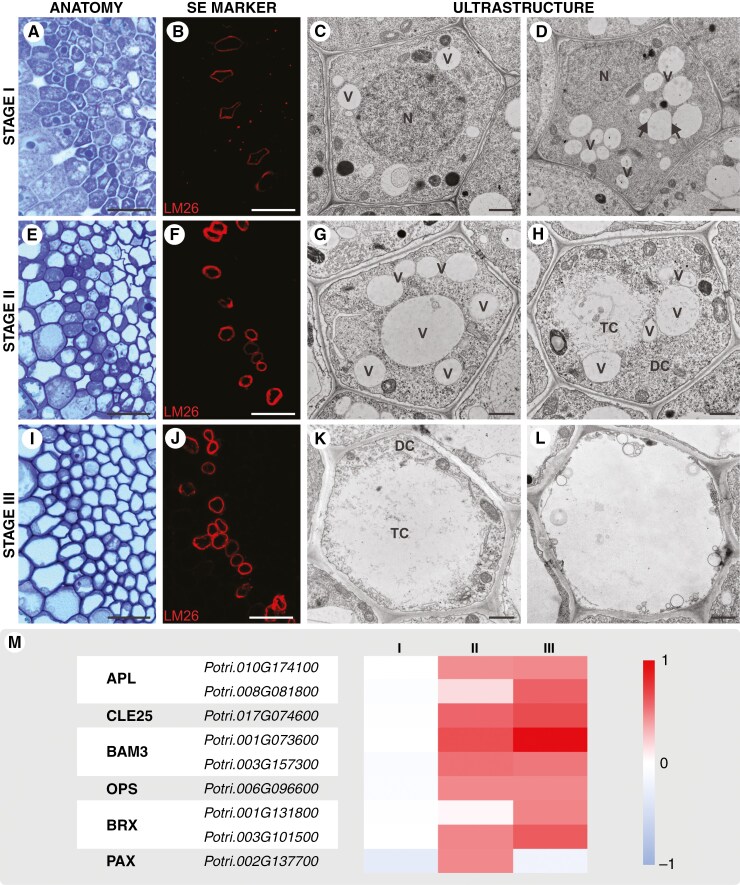
Developmental progression of metaphloem SEs in pioneer roots of *P. trichocarpa*. (A, E, I) Stages of phloem development (scale bars = 20 μm). (B, F, J) Localization of an SE cell wall marker (red fluorescence) detected by LM26 antibody (recognizing a 1,6-galactosyl substitution in 1,4-β-d-galactans) across stages I–III of phloem development (scale bars = 20 μm); (C, D, G, H, K, L) TEM micrographs showing the transformation from SE precursors to mature functional sieve tubes. N, nucleus; V, vacuole; arrows indicate merging vacuoles, two phases of cytoplasm with different mass density: TC, electron-translucent cytoplasm; DC, electron-dense cytoplasm (scale bars = 1 μm). (M) Relative expression of genes encoding different phloem-specific markers.

The study’s experimental design focused on identifying SE development stages characterized by structural changes at both tissue and cellular levels. Multi-methodological approaches, including anatomical and histochemical analyses, were employed to correlate these stages with ultrastructural observations of differentiating cells (Fig. 2). The analysis proceeded from mature SEs towards the root tip, revealing that phloem in the primary structure of *P. trichocarpa* pioneer root forms a complex cellular system within the stele, situated just beyond the pericycle. Differentiation from procambial tissue follows a centripetal, radial pattern typical for angiosperms, starting with rapid maturation of protophloem SEs, connected to two phloem pole pericycle cells and two companion cells (CCs). The first metaphloem SE is located between protophloem SE and CCs, but the next cells mature unevenly in the elongation zone. Anatomical observations (Fig. 2A, E, I) and specific immunostaining of SE cell walls with the LM26 antibody (Fig. 2B, F, J) facilitated the distinction of three phloemogenesis developmental stages. Metaphloem SEs were selected for detailed analysis of cellular content changes. Criteria for stage differentiation included the presence and structure of the nucleus, vacuole size changes, autophagic activity and cytoplasmic clearance at the cell centre. Initially, SE precursors displayed a large nucleus (Fig. 2C), with early differentiation marked by an increasing number of small vacuoles, pushing the nucleus to a peripheral position (Fig. 2D). Subsequently, vacuoles enlarged and merged, forming vacuoles of varied sizes (Fig. 2G), some of which degenerated. This led to partial degradation of the protoplast, resulting in the separation of two distinct cytoplasmic phases: electron-dense cytoplasm with organelles and electron-translucent cytoplasm with fewer ribosomes. Progressed SE development featured a predominant translucent cytoplasm contributing to the formation of a lumen for substance transport, facilitated by internal vacuolization or the release of vacuolar contents into the lumen (Fig. 2H). During SE maturation, the dense cytoplasm with organelles remained adjacent to the wall (Fig. 2K). The final stage of development saw the breakdown of the boundary between the two cytoplasmic phases due to extensive lytic vacuole activity and protoplast degeneration, culminating in the formation of an enucleated cell with minimal organelle content (Fig. 2L, Supplementary Data Fig. S6A). Molecular analyses of genes encoding phloem-specific proteins (Fig. 2M) were conducted for root segments corresponding to the identified phloemogenesis stages (I–III, Fig. 2A–L), confirming the experimental approach’s validity. Screening for phloem-specific genes, as identified in the model species *A. thaliana*, included *ALTERED PHLOEM DEVELOPMENT* (*APL*), *CLAVATA3/ESR-RELATED 25* (*CLE25*), *BARELY ANY MERISTEM 3* (*BAM3*), *OCTOPUS* (*OPS*) and *BREVIS RADIX* (*BRX*) interacting with *PAX* (*PROTEIN KINASE ASSOCIATED WITH BRX*) (Supplementary Data Table S1). Comparative genomic analysis revealed that these genes possess one or more potential orthologues in the *P. trichocarpa* genome. The expression profiles of almost all selected genes exhibited a statistically significant increase in stages II and III with the exception of a *PAX* orthologue, which showed increased expression in stage II followed by a decrease in stage III (Fig. 2M).

Conspicuous features were ascribed to each cell type of mature phloem in *P. trichocarpa* root. Sieve elements were characterized by homogeneous cytoplasm, with the only specific cellular components observed near the cell wall being mitochondria, endoplasmic reticulum, plastids, Golgi apparatus and various vesicles. Metaphloem SEs predominantly exhibited a regular, often pentagonal cross-sectional shape (Fig. 2L, Supplementary Data Fig. S6A), in contrast to narrower, rapidly degenerating protophloem SEs (Supplementary Data Fig. S6B). Phloem parenchyma cells appeared rounded in section, with a thin cell wall and large central vacuole (Supplementary Data Fig. S6C), potentially exerting pressure on neighbouring SEs and occasionally distorting their shape. Companion cells were distinguished by smaller size and their dense cytoplasm (Supplementary Data Fig. S6D), though they were relatively infrequent in the pioneer roots of *P. trichocarpa*.

### Autophagic events

**Fig. 3.**
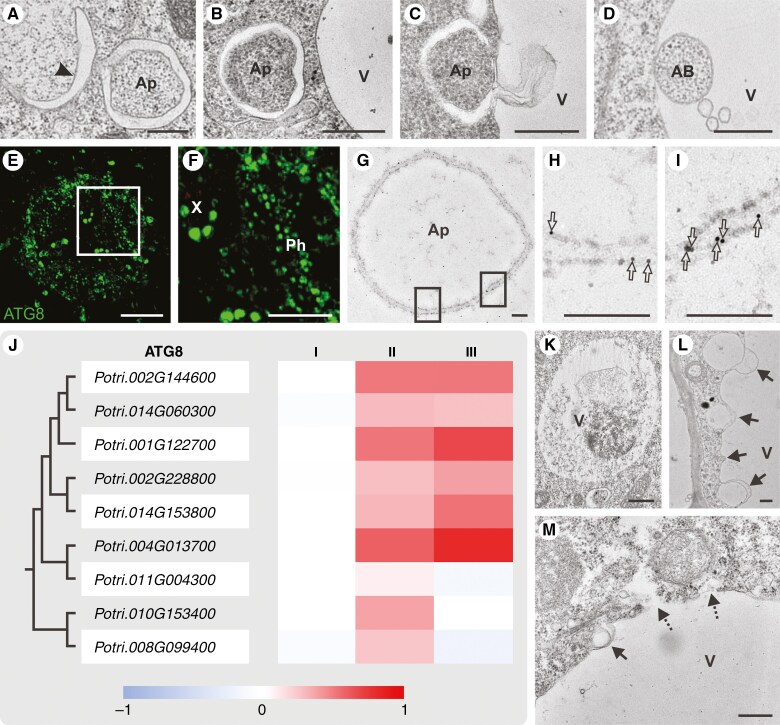
Autophagic activity during SE development in *P. trichocarpa* roots. (A–J) Macroautophagy processes. Ap, autophagosome. (A) Phagophore formation (arrowhead). (B) Autophagosome development. (C) Autophagosome fusion with vacuoles. (D) Release of autophagic bodies (AB). (E, F) Immunolocalization of ATG8 protein within the root vascular cylinder, in both xylem (X) and phloem (Ph). (G–I) Autophagosome with ATG8 immunolabelling (empty arrows in magnifications). (J) Phylogenetic tree and relative expression levels of ATG8 protein-encoding genes across three SE development stages. (K) Lytic vacuole rupture showing degenerating cytoplasmic content. (L, M) Microautophagy initiation through cytoplasmic invagination, forming buds (arrows) that incorporate cytoplasm fragments or vesicles. (M) Disintegrated vacuole with tonoplast permeabilization resulting in content leakage (dashed arrows). V, vacuole. Scale bars: A–D, K–M = 500 nm, E, F = 50 μm, G–I = 200 nm.

Ultrastructural analysis of phloemogenesis stages revealed simultaneous engagement of micro- and macroautophagy in SE differentiation. Early stages featured numerous vacuoles of varying shapes and sizes, some undergoing fusion via tonoplast merging (Fig. 2D). Differentially shaped and sized autophagic structures, such as phagophores (Fig. 3A), autophagosomes delivering cargos (Fig. 3A, B), and their fusion with vacuoles (Fig. 3C), were documented in many SEs. This resulted in a variety of autophagic bodies within vacuoles, differing in size and content density (Fig. 3D). Immunohistochemical methods confirmed autophagy in developing SEs, with ATG8 epitopes detected in both xylem and phloem conductive elements (Fig. 3E, F). Instead, ultrastructural localization identified double-membrane vesicles, with ATG8 present in both the inner and the outer membrane, as autophagosomes (Fig. 3G–I). The expression analysis of genes encoding ATG8 proteins revealed distinct expression profiles correlating with phloemogenesis stages, with five genes showing stage-specific, significant increases in expression (Fig. 3J). One gene (*Potri.014G060300*) exhibited increased expression in stages II and III relative to stage I, though expression in stage III was slightly reduced compared with stage II. Three *ATG8* orthologues (*Potri.011G004300*, *Potri.010G153400* and *Potri.008G099400*) displayed distinct expression patterns, with a statistically significant increase in stage II followed by decrease in stage III. Two of these genes are closely related and distinct from the other *ATG8* orthologues based on phylogenetic analysis. Only one gene (*Potri.011G004300*) showed a statistically significant change in expression exclusively in stage II (Fig. 3J).

From the early stages of SE development, small lytic vacuoles were present and became increasingly frequent as phloemogenesis progressed. These vacuoles collapsed, digesting material, including membrane fragments, and varied in density (Fig. 3K). In addition to macroautophagy, microautophagy was observed, characterized by tonoplast bending that led to inclusions or invaginations in the form of tubes or buds that incorporate cytoplasm portions or vesicles into the vacuole (Fig. 3L, M). These processes contributed to vacuole biogenesis and enlargement. Subsequent vacuole disintegration, due to partial tonoplast permeabilization, resulted in the release of vacuolar contents (Fig. 3M). However, this loss of tonoplast integrity did not lead to complete protoplast degradation; vacuoles expanded in volume but did not dominate the cellular space (Fig. 2C, D, G, H). Therefore, vacuolar degeneration led only to localized degradations and a gradual reduction in cytoplasmic density within SEs (Fig. 2H, K). In mature SEs, vacuoles were absent (Fig. 2L, Supplementary Data Fig. S2A), contrasting with the numerous smaller vesicles scattered and positioned adjacent to the cell wall.

### Mechanisms of selective autophagy involved in organelle removal

**Fig. 4.**
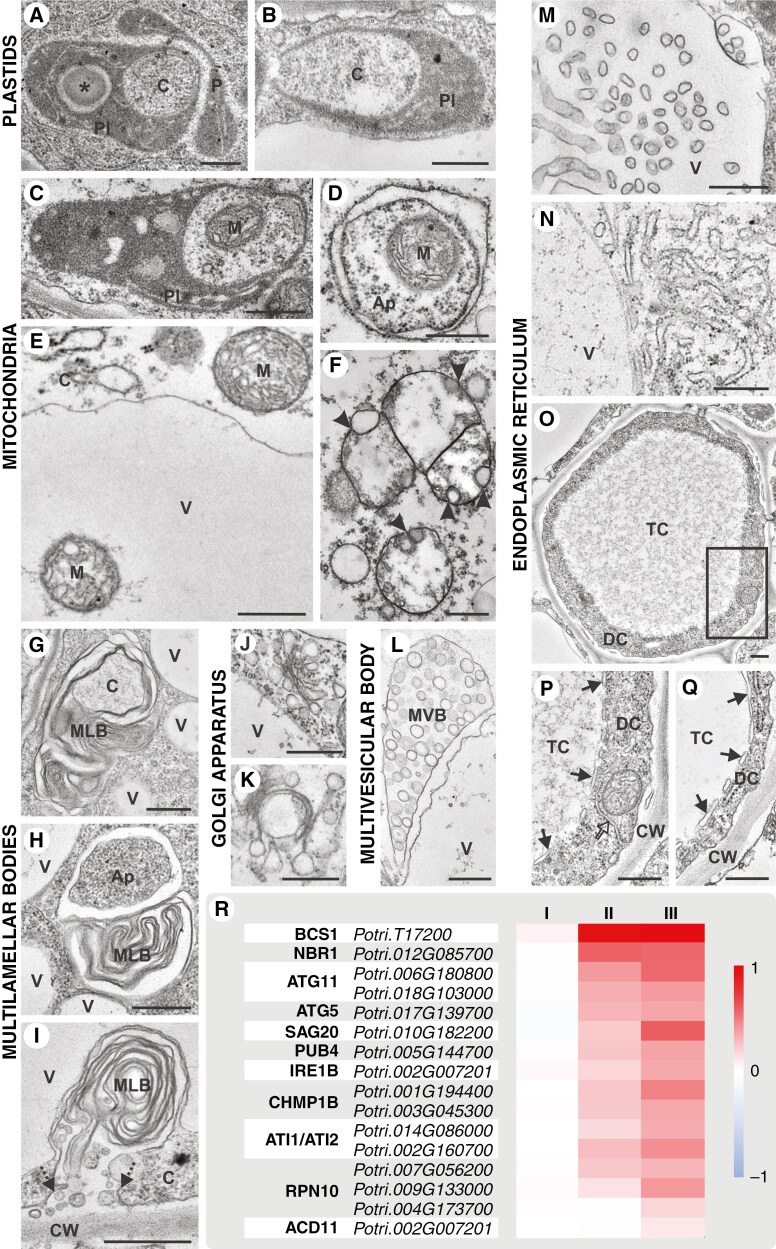
Selective organelle removal during SE development in the pioneer root of *P. trichocarpa*. (A) Curled plastid (P) and an autophagic plastid - plastolysome (Pl) with a cytoplasm portion (C); asterisk indicates starch grain. (B) Plastolysome (Pl) engulfing a cytoplasm portion (C). (C) Plastolysome (Pl) containing a mitochondrion (M). (D) A mitochondrion (M) in an autophagosome (Ap). (E) Digestion of a mitochondrion (M) within a vacuole. (F) Fusion and outer membrane proliferation (arrowheads) of mitochondrion-derived vesicles. (G) Cytoplasm portion (C) engulfed by excessive membranes forming a multilamellar body (MLB). (H) MLB fusion with an autophagosome (Ap). (I) MLB excretion out to the apoplast, with dashed arrows indicating the cell membrane. (J) Cisternae and vesicles of Golgi apparatus. (K) Cisternae of Golgi apparatus with autophagosome-like structure. (L) Multivesicular body (MVB). (M) Vacuolar incorporation of excessive ER-derived membranes. (N) ER fragmentation around the vacuole. (O, P) Formation of an ER-derived membrane system (arrows in magnification) demarcating denser cytoplasm (DC) with organelles, some of which are surrounded by another type of ER (empty arrow), and (Q) electron-translucent cytoplasm (TC) in the developing SE centre. V, vacuole; C, cytoplasm; CW, cell wall. Scale bars = 500 nm. (R) Relative expression of genes associated with selective autophagy and protoplast degradation across three phloemogenesis stages.

The degradation of individual organelles in SEs occurred in a coordinated and specific manner, following a defined pattern that underscores the selective nature of these degradation pathways. Initially, plastids in SEs during the onset of phloemogenesis exhibited a very dense stroma, making the plastid interior indistinguishable from its membranes, with only plastoglobules being clearly visible. As phloemogenesis progressed, some plastids began producing numerous starch grains, while others underwent curling (Fig. 4A) to engulf a portion of the cytoplasm. This resulted in the formation of autophagic plastids known as plastolysomes (Fig. 4A–C) characterized by multiple layers, resembling multilamellar bodies (MLBs). Mature SE-specific plastids appeared large and swollen, containing sparse plasma and starch grains, being anchored to the wall by cisternae of sieve endoplasmic reticulum (SER).

Early in SE differentiation, numerous mitochondria were observable; however, redundant mitochondria were selectively removed through various mechanisms. Their presence was noticed within plastolysomes (Fig. 4C) and autophagosomes (Fig. 4D). In the final stages of SE maturation, some mitochondria were digested within vacuoles (Fig. 4E), while others exhibited a sparse matrix and progressive disintegration of inner membranes, leading to the formation of single-membrane vesicles. These vesicles either broke down or remained in mature SEs close to the cell wall. On the other hand, the outer membrane of disintegrated mitochondria underwent proliferative activity and fusion (Fig. 4F), forming multivesicular bodies (MVBs). Surviving SE-specific mitochondria were anchored to the cell wall by SER cisternae, being more rounded, and exhibiting swollen inner membranes.

Throughout phloemogenesis, excessive and dysfunctional membranes in SE incorporated some of cytoplasm (Fig. 4G) or were removed within vacuoles (Fig. 4H, I). This processes led to the formation of numerous MLBs, potentially originating from ER degradation or the multilayered vesicles formed from plastolysomes. Multilamellar bodies often fused with autophagosomes near vacuoles (Fig. 4H) and were frequently present within the vacuolar lumen. Mature MLBs, containing only membranes, were expelled from protoplasts by excretion out to the apoplast (Fig. 4I). During SE differentiation, numerous cisternae and vesicles of the Golgi apparatus were observed. Subsequently, the number of cisternae decreased, while vesicle numbers increased (Fig. 4J). By the end of phloemogenesis, most dictyosomes had irregular organization, fewer cisternae and no vesicles. The cisternae had entirely fragmented, although some curved to form an autophagosome-like structure and persisted in mature SEs (Fig. 4K). The degeneration of mitochondria, Golgi apparatus activity, accumulation of fragmented membranes, and autophagic structures led to the formation and fusion of numerous vesicles, aggregation of MVBs (Fig. 4L) and cytoplasmic vesiculation.

The ER architecture in SEs was highly dynamic during phloemogenesis and played a crucial role in organellar compartmentalization. Initially, long, regular sheets of rough ER were observed. As development progressed, the ER often lost its regular structure, becoming dilated and fragmented. These swollen ER fragments formed a network of tubes, cisternae or vesicles. Excess membranes were either massively removed within vacuoles (Fig. 4M), contributing to a reduction in cytoplasmic density at the SE centre, or partially remained around these vacuoles (Fig. 4N). Not only did internal vacuoles form the lumen, but it was also enlarged by surrounding vacuoles releasing their contents into it due to tonoplast damage (Fig. 2H). Other ER-derived membranes served to compartmentalize these processes, leading to the formation of phases with different cytoplasm densities. As SE development progressed, electron-translucent cytoplasm expanded to a point almost occupying the intervening space between cell walls in comparison with the significant decrease in the case of electron-dense cytoplasm (Fig. 4O, P, Fig. 2K). A different type of ER was noticed surrounding organelles near the cell wall (Fig. 4Q). Both rough and smooth ER, exhibiting various structures, was present simultaneously during SE development. In mature SEs, narrow and short ER sheets with vesicles were evident, or massive ER aggregates and ER cisternae probably fused with many vesicles and SE-specific organelles, anchoring them to the cell wall.

To understand the persistence of certain organelles in mature SEs and the degradation of others, it was essential to examine also expression levels of genes encoding proteins involved in autophagy and PCD processes. Selected genes (Supplementary Data Table S1) known for their functions in these processes in *A*. *thaliana* include *BCS1* (*CYTOCHROME BC1 SYNTHESIS*), *NBR1* (*NEXT TO BRCA1 GENE 1*), *ATG5* (*AUTOPHAGY-RELATED GENE 5*), *ATG11* (*AUTOPHAGY-RELATED GENE 11*), *SAG20* (*SENESCENCE ASSOCIATED GENE 20*), *PUB4* (*PLANT U-BOX 4*), *IRE1B* (*INOSITOL REQUIRING 1-1*), *CHMP1B* (*CHARGED MULTIVESICULAR BODY PROTEIN/CHROMATIN MODIFYING PROTEIN 1B*), *ATI1* and *ATI2* (*ATG8-INTERACTING PROTEIN 1/2*), *RPN10* (*MULTIUBIQUITIN CHAIN BINDING PROTEIN 1*) and *ACD11* (*ACCELERATED CELL DEATH 11*). Genomic analysis was conducted to identify potential orthologues (one to three per gene) in the *P. trichocarpa* genome. The expression levels of these genes were assessed across phloem developmental stages, revealing a statistically significant increase for the majority of the tested genes. The exceptions were the *Potri.006G051800* gene (*ACD11* potential orthologue) and *Potri.004G173700* (one of the *RPN10* orthologues), which showed a slight increase in expression only in developmental stage III. Conversely, the expression of *Potri.T17200*, identified as a potential orthologue of *BCS1*, was significantly higher in developmental stages II and III compared with other tested genes (Fig. 4R).

### Enucleation

**Fig. 5.**
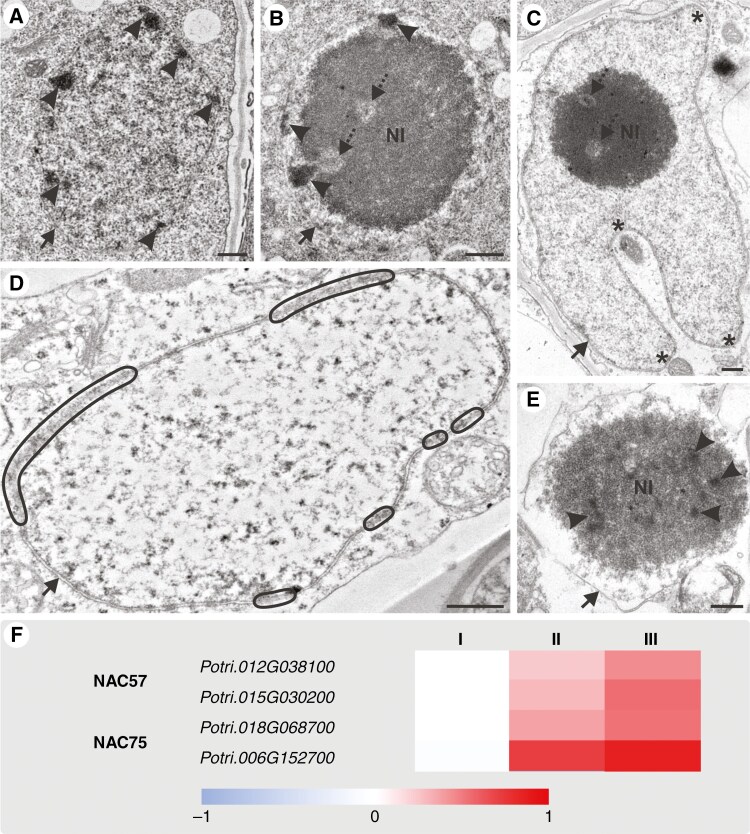
Nuclear transformation and enucleation during SE development in *P. trichocarpa* roots. (A–C, E) Changes in nuclear plasma density of nucleus or nucleolus (arrowheads indicate increases, dashed arrows indicate decreases). (C) Nucleus with abnormal structure featuring blebs and invaginations (asterisks). (D) Nucleus with sparse content and nuclear envelope with ruptures (outlined). (E) Final stage of SE development showing nucleolus disintegration and nuclear envelope fragmentation; NL, nucleolus. Arrows in (A–E) indicate nuclear envelope. Scale bar = 500 nm. (F) relative expression levels of various SE-specific genes encoding transcription factors involved in enucleation during three stages of phloemogenesis.

Progressive disintegration of the nucleus led to its degradation, a hallmark of SE differentiation, commencing in the final maturation stage. Initially, the nucleus was rounded (Fig. 5A) and located in the central position of the SE (Fig. 2C). Throughout development, the nuclear plasma underwent various changes, including the appearance of large nucleoli and alterations in plasma density, indicative of potential chromatin condensation or dispersion (Fig. 5B, C, E). The nucleus also lost its regular structure and dominant position within the cell (Fig. 2D). Advanced SE development featured an abnormally shaped nucleus with blebs and invaginations, surrounded by thin cytoplasm and containing sparse plasma. However, the nuclear envelope and nucleolus remained intact (Fig. 5C). Subsequently, the nuclear envelope became ruptured, and nuclear plasma with significantly decreased density was observed (Fig. 5D). The final stages involved nucleolus disintegration and nuclear envelope fragmentation (Fig. 5E), resulting in mature SEs devoid of a nucleus (Fig. 2L, Supplementary Data Fig. S6A). TUNEL assays confirmed genetically programmed chromatin fragmentation in nuclei of developing SEs, with negative controls showing no detectable signal (Supplementary Data Fig. S5). Several transcription factors associated with phloem development and enucleation were investigated, including NAC proteins, with NAC057 and NAC075 being specific to SEs. Genomic comparison analysis identified two potential orthologues of these NAC-encoding genes in *P. trichocarpa*. The relative expression of these genes significantly increased in subsequent root developmental stages, with one potential orthologue of *NAC075* (*Potri.*006G152700) exhibiting markedly higher relative expression compared with other tested genes (Fig. 5F).

## DISCUSSION

Plants are initially equipped with a genetic programme that enables them to selectively remove redundant or damaged organelles, cells, tissues, and even entire organs via PCD mechanisms. However, the activation of PCD pathways rarely culminates in the death of the entire organism. Emerging evidence highlights autophagy as a critical regulator of PCD in plants (Kabbage *et al.*, 2017). Interestingly, mature SEs inhibit PCD-specific mechanisms, illustrating the meticulous regulation of cytoplasmic degradation in SEs. It is a new idead that this regulation is governed primarily through autophagy. This process, when directed specifically towards the degradation of macromolecules, proteins, aggregates or structures, is termed ‘selective autophagy’ (Floyd *et al.*, 2012). Sieve element differentiation shares similarities with autophagic cell death, characterized by an increase in vacuole size and the engulfment of organelles into the vacuole via micro- and macroautophagy, followed by organelle degradation and eventual vacuole lysis, yet without proceeding to complete cellular demise. This study reveals that autophagy indicators at the onset of various phloemogenesis stages are remarkably specific, unfolding within a well-defined spatiotemporal framework for the selective elimination of organelles and cytoplasmic clearing in SEs. Macroautophagy in developing SEs is confirmed by observations of phagophores and autophagosomes. These structures arise from the endoplasmic reticulum, Golgi apparatus, mitochondrial membranes, or directly from lytic prevacuoles with collapsed membranes (Li and Vierstra, 2012; Chan and Tang, 2013; van Doorn and Papini, 2013; Zhuang *et al.*, 2017) as seen in examined SEs. In the pioneer roots of *P. trichocarpa*, ATG8 proteins, implicated in macroautophagy, were present during phloemogenesis. Autophagy-related (ATG) proteins are known for their role in the activation and formation of autophagic structures, cargo recruitment, autophagosome transport, and fusion with vacuoles (Jacomin *et al.*, 2018; Liu *et al*., 2018; Yoshimoto and Ohsumi, 2018). Unique ATG8 is the sole protein involved in all these stages (Nakatogawa *et al.*, 2007; Xie *et al.*, 2008; Klionsky and Schulman, 2014). ATG8s located on the outer membrane binds to autophagy adaptors, facilitating autophagosome delivery and fusion with vacuoles (Ketelaar *et al.*, 2004; Michaeli *et al.*, 2014; Brillada *et al.*, 2018; Yoshimoto and Ohsumi, 2018). Crucially, inner membrane ATG8 within the phagophore acts as a driver of selective autophagy (Kellner *et al.*, 2017), collaborating with receptors that recognize degradation targets (Noda *et al.*, 2010) and are known as selective autophagy markers (Islam *et al.*, 2019; Ran *et al.*, 2020). This specificity renders macroautophagy a fundamental process in terms of autophagic selectivity (Floyd *et al.*, 2012; Gatica *et al.*, 2018; Marshall *et al.*, 2022; Rasmussen *et al.*, 2022). Therefore, the upregulated expression of genes encoding selective autophagy receptors that associate with ATG8 localized in the phloem underscores the role of selective autophagy in SE development. This study points to the operation of tightly regulated pathways for organelle removal. The transition of SEs from meristematic cells is governed by a phloem-specific genetic framework, with notable changes in the expression of ATG8-encoding genes. Localization of ATG8 protein in developing SEs across diverse species and the proposed differential roles of various ATG8 isoforms in autophagy during PCD or selective autophagy (Michalak *et al.*, 2024) highlight the complexity of this regulatory mechanism. Recent single-cell sequencing analysis of *A. thaliana* root has revealed that higher expression levels of some *ATG*s occurred in phloem initial cells, differentiating phloem or during a complete timeline of phloem development (Wendrich *et al.*, 2020). In another study of *A. thaliana* root, the expression of *ATG8B* and *ATG8D* was identified as specific to phloem cells (Roszak *et al.*, 2021). Additionally, *ATG* genes are expressed in both the primary and secondary structure of roots and stems (Wojciechowska *et al.*, 2019). The evolutionary conservation of this process further affirms the role of RPN10 in autophagy during SE development. The ubiquitin receptor RPN10, previously identified as an autophagy receptor that targets ubiquitylated proteasomes for degradation by binding to ATG8 (Marshall *et al.*, 2022), emphasizes the selectivity of RPN10-mediated degradation (Wen and Klionsky, 2016). Similarly, *RPN10* expression increases in succesive SE developmental stages in *A. thaliana* roots (Wendrich *et al.*, 2020), which supports its function in selective autophagy during phloemogenesis. Thus, these autophagic events may orchestrate the structuring of SEs as cells with minimal content, yet capable of substance transport, delineating a sophisticated regulatory network underlying this developmental process.

Taken together, the results of this study provide new evidence that links microautophagy and macroautophagy to the selective degradation of organelles during the development of phloem conductive cells. These processes can occur concurrently and are pivotal in vacuole biogenesis (van Doorn and Woltering, 2005; Inoue *et al.*, 2006; Yano *et al.*, 2007; Machado and Rodrigues, 2019). Additionally, vacuoles may expand considerably through the amalgamation of multiple vacuoles (van Doorn *et al.*, 2015), a phenomenon critical to mega-autophagy. In the xylem, this leads to extensive protoplast degradation and autolysis, facilitated by tonoplast permeabilization or rupture (van Doorn and Woltering, 2005, 2010; van Doorn and Papini, 2013; Wojciechowska *et al.*, 2021), enabling the interaction of accumulated hydrolytic enzymes with organelles. However, in SEs, numerous organelles seemed to remain functional after vacuole collapse, suggesting that tonoplast disintegration does not compromise the surviving cell contents. Notably, only a limited number of inclusions are observed immediately prior to or at the onset of vacuole rupture, challenging the likelihood of tonoplast rupture and subsequent release of large quantities of hydrolytic enzymes. This raises the possibility that the remaining organelles do not undergo degradation, a scenario critically assessed and refuted in this study. In the developing caryopsis, the central vacuole and cytosol of differentiating SEs exhibit slight acidification following tonoplast rupture (Yang *et al.*, 2015). Intriguingly, barley vacuolar aspartic proteinase (phytepsin homologous to animal cathepsin D) is detectable in roots during both TE autolysis and partial SE autolysis (Runeberg‐Roos and Saarma, 1998). The vacuole’s role in facilitating cytoplasmic degeneration to prepare SEs for substance transport has been proposed previously (Wang *et al.*, 2008). Initially, the vacuole mediates microautophagy and eventually disintegrates, releasing proteases. The enzymatic activity might be moderated by the vacuole’s mildly acidic sap, thus not proceeding PCD. It has been hypothesized that cytoplasmic fragments containing organelles could be isolated through tonoplast and plasma membrane fusion following the central vacuole’s degradation (Yang *et al.*, 2015). However, this study reveals the absence of such vacuoles during SE development, with various sizes potentially degenerating earlier in the differentiation process, leading to localized cytoplasmic degradation. Contrarily, mature SEs lack vacuoles or tonoplast fragments aligning with observations in *A. thaliana* roots where multiple small vacuoles are present during SE development, rather than a single dominant one (Furuta *et al.*, 2014). These findings affirm that vacuolar disintegration and its role in reducing cytoplasmic content in SEs are as meticulously regulated as macroautophagy. Plants might possess mechanisms to either empty the vacuole, including hydrolytic enzymes, prior to rupture or to mitigate the enzymes’ activity, thereby preserving numerous organelles. In *P. trichocarpa* root SEs, organelles are maintained proximal to the cell wall, safeguarded against vacuolar degradation of the entire protoplast by the SER. The ER-derived membrane system undergoes specific transformations during SE differentiation, forming structures that possibly isolate degradative processes and organelles, and subsequently leading to phases of electron-translucent and electron-dense cytoplasm. The distribution of ER throughout the SE, demonstrated in *Nicotiana tabacum*, implies similar SER functionalities (Knoblauch and Peters, 2010). However, this study documents the simultaneous presence of various ER types during SE development, with the membranes responsible for organellar compartmentalization disappearing by the end of phloem development. Only minor ER fragments or aggregates and cisternae that anchor organelles close to the cell wall persist in mature SEs. Furthermore, the ER may contribute to the autolysis of SE cytoplasmic content (Oparka *et al.*, 1981). The survival of mitochondria and starch-filled plastids might also counteract PCD. Plant cells receiving a developmental death signal can be rescued before reaching the point of no return during autophagic PCD if even a minimum number of functional, albeit degenerated, mitochondria and plastids exist (van Doorn, 2005; van Doorn and Woltering, 2005). Moreover, the nucleus undergoes degradation at the final stage of SE maturation, as previously verified by molecular studies of *A. thaliana* roots (Roszak *et al.*, 2021). Post-enucleation, mitochondria, plastids and ER fragments, along with vesicles of diverse origins, remain functional with conventional architectures, supported by CCs to maintain SE viability. Autophagy plays a pivotal role in the selective degradation of cellular components in SEs, and the products of this process, resulting from the digestion of autophagic bodies within vacuoles, may also be repurposed by the cell (van Doorn and Woltering, 2010; Minina *et al.*, 2014). These recycled nutrients could further support SE survival, highlighting another vital role of selective autophagy in phloemogenesis.

The organelle reduction within SEs is attributed to autophagic processes and inherent self-degeneration. This study also reports an upsurge in the expression levels of genes that are potential orthologues of *A. thaliana ATI1* and *ATI2* (*ATG8-INTERACTING PROTEIN 1* and *2*). During phloem development in *A. thaliana* roots expression of these genes is high as well, especially in phloem initial cells (Wendrich *et al.*, 2020). ATI1/2 has been previously identified as a polypeptide that binds to the ATG8f protein (Honig *et al.*, 2012). Moreover, ATI1 interacts with specific plastid proteins, facilitating organelle degradation within vacuoles (Michaeli *et al.*, 2014). Gene expression analysis indicated an increase in the expression level of the *PUB4* orthologue in *P. trichocarpa* across successive phloem developmental stages, which confirms previously obtained data for phloem in *A. thaliana* roots (Wendrich *et al.*, 2020). The PUB4 pathway is linked to chloroplast-targeted autophagy (Kikuchi *et al.*, 2020). Certain plastids, mirroring macroautophagic structures, have been documented in developing SEs. These autophagic plastids engulf portions of the cytoplasm and transform into plastolysomes, which are then delivered to the vacuole. Degradation of sequestered cargo and plastolysome inner membranes promotes the formation of MLBs that are expelled into the apoplast (van Doorn and Papini, 2013; Parra-Vega *et al.*, 2015) as another selective removal process. This unique mechanism has been previously observed in limited developmental contexts, such as embryogenic microspores (Parra-Vega *et al.*, 2015) and leaf secretory cavities (Zhou *et al.*, 2014). Acid phosphatase, indicative of autophagic activity, was detected in plastolysomes during petal development (van Doorn *et al.*, 2011) and in senescent embryo-suspensor cells (Nagl, 1977; Gärtner and Nagl, 1980). Thus far, autophagosome and MLB fusion has only been observed in epithelial cells (Li *et al.*, 2022). Observations of these structures in developing SEs have indicated that MLB removal is also autophagy-related in plants. CHMP1B, another factor linked to plastid degradation, promotes phagophore maturation and aids in the seamless secretion of plastids or their fragments into autophagosomes, leading to the formation of MLBs such as rubisco-containing bodies (RCBs) and small starch granule-like (SSGL) bodies (Wan and Ling, 2022). In *A. thaliana* roots, phloem-specific expression of *CHMP1B* was relatively higher, especially in phloem initials and SEs (Wendrich *et al.*, 2020). In the roots of *P. trichocarpa*, the gene expression level of *CHMP1B* was found to increase through subsequent phloem developmental stages with numerous MLBs observed in developing SEs. Different autophagy types mediate mitochondrial removal, with some mitochondria undergoing degeneration that leads to cytoplasmic vesiculation or their transformation into vesicles and MVBs, mechanisms akin to those observed in virus-induced PCD (Hatta and Ushiyama, 1973; Rubino *et al.*, 2014). Increased expression of *ATG11* in phloem development stages, known for reducing mitochondrial size for autophagosome encapsulation (Mao *et al.*, 2013), supports the autophagosomal removal of mitochondria. It matches the higher relative expression of *ATG11* during phloem development, especially in phloem initials and CCs in *A. thaliana* roots (Wendrich *et al.*, 2020), which was confirmed in phloem-specific scRNA-seq analysis (Roszak *et al.*, 2021). The presence of MVBs may also relate to autophagic mechanisms responsible for the degeneration of ER fragments and the Golgi apparatus (Cui *et al.*, 2018). However, in SEs dictyosomes disintegrated into an autophagosome-like structure, as observed previously in senescing petals (Nabipour Sanjbod *et al.*, 2023). The rising expression of *IRE1B* during phloem development corroborates ER autophagy (Liu *et al.*, 2012; Bao *et al.*, 2018). This has been observed in this research and in the single-cell sequencing of *A. thaliana* roots as well (Wendrich *et al.*, 2020). In the degenerative processes within SEs, the disposal of membranes must be controlled, leading to the formation of MLBs not only from plastid or plastolysome degradation, but also from the presence of surplus membranes. In plant cells, excessive membranes are organized during differentiation (Sandor *et al.*, 2021). Interestingly, the expression of *ACD11*, which is gene-specific to PCD, was lower for the first two stages of phloemogenesis in *P. trichocarpa* root; however, in the root of *A. thaliana*, *ACD11* expression was also identified in the phloem (Roszak *et al.*, 2021), especially in CCs (Wendrich *et al.*, 2020). Enucleation, as the last stage of SE development, was connected to NAC45 and NAC86 transcription factors as final drivers of this process in *A. thaliana* roots (Furuta *et al.*, 2014). However, these data have not been confirmed in single-cell analysis. The expression of *NAC45* was poorly visible in only a few phloem procambium cells and high expression of *NAC86* was noticed for only one SE (Wendrich *et al.*, 2020). Considering this, it was necessary to find other enucleation markers specific for SEs. In *A. thaliana*, NAC57 has been revealed as highly specific for SEs and NAC75 as characteristic of phloem development in single-cell transcriptomics of all roots (Wendrich *et al.*, 2020) and phloem developmental stages (Roszak *et al.*, 2021). Potential orthologues of *NAC57* and *NAC75* in *P. trichocarpa* exhibited an increasing expression in roots during phloemogenesis.

In summary, the reprogramming of SE fate towards phloemogenesis is a complex and intriguing process that involves a variety of degradation mechanisms derived from the developmental switch. Historical studies dating back to Hartig in 1937 have primarily focused on the cellular composition of mature sieve tubes (Evert, 1977; Eleftheriou, 1986), with a lack of detailed ultrastructural observations tracing the transformation from meristematic cells to mature SEs. Although PCD-like symptoms have been noted in SEs, which remain viable, this underscores the unique nature of phloemogenesis in annual plants like *Triticum aestivum* (Wang *et al.*, 2008; Yang *et al.*, 2015) and *A. thaliana* (Furuta *et al.*, 2014). The extent and nuances of this process may vary across species. Numerous questions about the cellular mechanisms underpinning phloemogenesis and organelle degradation selectivity remain. SE-specific traits like *in vivo* protoplast acidification, individual organelle viability and changes in an organelle number for each developmental stage need to be examined. Despite limited explicit literature, the significance of this research area is widely recognized. This study provides a comprehensive narrative of metaphloem SE development in *P. trichocarpa* pioneer roots, highlighting selective autophagy as a crucial mechanism for the precise elimination of cytoplasmic components. Phloemogenesis emerges as a complex interplay between degradation and survival, offering a rich yet challenging, model for exploring selective autophagy mechanisms.

## SUPPLEMENTARY DATA

Supplementary data are available at *Annals of Botany* online and consist of the following.

Table S1: list of genes selected for RT–qPCR analyses based on the Phytozome database (https://phytozome-next.jgi.doe.gov/). Figure S1: relative expression of selected gene s in *P. trichocarpa* pioneer roots during phloemogenesis. The ID of each gene and the name of the encoded protein are indicated at the bottom of the histogram. Figure S2: transmitting light micrographs (LM) and negative control for localization of an SE cell wall marker (red fluorescence) detected by LM26 antibody (recognizing a 1,6-galactosyl substitution in 1,4-β-d-galactans) within the root vascular cylinder in pioneer root of *P. trichocarpa*. Scale bars = 100 μm. Figure S3: transmitting light micrographs (LM) and negative control for localization of ATG8 protein within the root vascular cylinder, in both xylem (X) and phloem (Ph) in pioneer root of *P. trichocarpa*. Scale bars = 50 μm. Figure S4: representative images of the negative control, which omitted the primary antibody anti-ATG8; no immunogold labelling was detected in sieve elements of *P. trichocarpa* root. Scale bars = 500 nm. Figure S5: TUNEL assay in SEs of *P. trichocarpa* root. (A) Positive results (arrows) in developing SEs. (B) Corresponding nuclear identification (arrows) via DAPI staining. (C, D) Negative control, which omitted the TdT reaction mixture during incubation. Scale bars = 20 μm. (E, F) Positive control after DNase treatment of root sections shows degradation in all nuclei. Scale bars = 100 μm. Figure S6: phloem components within primary structure of *P. trichocarpa* pioneer root (TEM). (A) Metaphloem SE with a distinctive pentagonal outline. (B) Protophloem SE. (C) Phloem parenchyma cell. (D) Companion cell. Scale bars = 1 μm.

mcae195_suppl_Supplementary_Table_S1

mcae195_suppl_Supplementary_Figure_S1

mcae195_suppl_Supplementary_Figure_S2

mcae195_suppl_Supplementary_Figure_S3

mcae195_suppl_Supplementary_Figure_S4

mcae195_suppl_Supplementary_Figure_S5

mcae195_suppl_Supplementary_Figure_S6

## Data Availability

The data supporting the findings of this study are available within the paper and within its supplementary data published online. The raw data underlying this article are available in Repository for Open Data (RepOD) at https://repod.icm.edu.pl/dataset.xhtml?persistentId=doi:10.18150/XEREHK and can be accessed with DOI:10.18150/XEREHK.
