# Supplementary material for: Is autophagy always a death sentence? A case study of highly selective cytoplasmic degradation during phloemogenesis
Source: Ann Bot. 2024 Nov 5;135(4):681–96. doi: 10.1093/aob/mcae195 (PMC11904893; doi:10.1093/aob/mcae195)
Supplement: mcae195_suppl_Supplementary_Table_S1 [file mcae195_suppl_supplementary_table_s1.docx]

**Table S1.** List of genes selected for RT-qPCR analyses based on Phytozome database (<https://phytozome-next.jgi.doe.gov/>), HGs – housekeeping genes selected for the normalization of expression values.

|  | **protein with function** | **ID of encoding gene** | **sequences of gene-specific primer pairs** |
| --- | --- | --- | --- |
| **HGs** | actin | *Potri.001G309500* | F: 5′-GCCCAGAAGTCCTCTTCCAG-3′  R: 5′-AAGGGCGGTGATCTCCTTG-3 |
|  | ubiquitin | *Potri.005G096700* | F: 5′-AGGAACGCGTTGAGGAGAAG-3′  R: 5′-TATAAGCAAAAACCGCCCCTG-3′ |
| **PHLOEM DEVELOPMENT** | APL (MYB FAMILY TRANSCRIPTION FACTOR; required for several aspects of phloem development in the root) | *Potri.010G174100* | F: 5’-CATGCCGAACAACGACAAC-3’  R: 5’-AGCGCTGGCTTTATTTCATACA-3’ |
|  |  | *Potri.008G081800* | F: 5’-CATGTCCAACAATCATGACGAC-3’  R: 5’-CCAGATAACGAATCCAGATCAGT-3’ |
|  | CLE 25 (CLAVATA3/ESR-RELATED 25; the BAM1 and BAM3 receptor-kinases are likely receptors for CLE25; involved in regulation of cell differentiation and phloem development) | *Potri.017G074600* | F: 5’-GGAATCCTACCAAACCATGCA-3’  R: 5’-AATAGGATCAGGTCCATTGGG-3’ |
|  | BAM3 (BARELY ANY Meristem 3; regulates expression of genes encoding CLAVATA1-related receptor kinase-like protein involved in vascular strand development) | *Potri.001G073600* | F: 5’-GCAACAGCTGAATTATCTGTCAC-3’  R: 5’-ATCGGTCCACTTAAGCTACAG-3’ |
|  |  | *Potri.003G157300* | F: 5’-GCAACAGCTGAATTATCTCTCGT-3’  R: 5’-CATCTAACTTGTTGAGGTTTCCC-3’ |
|  | OPS (OCTOPUS; a polarly localised membrane-associated protein that regulates phloem differentiation) | *Potri.006G096600* | F: 5’-CCAGCGTCAATGCTAATATTGG-3’  R: 5’-GTTTCGTGAACTGGCTCTG-3’ |
|  | BRX (BREVIS RADIX; a transcription factor being key regulator of cell proliferation and elongation in the root; modulates auxin flux through developing protophloem sieve elements (PPSEs) while interacting with PAX, thereby timing PPSE differentiation) | *Potri.001G131800* | F: 5’-GGTTCAAGTGGCTATATGGGA-3’  R: 5’-CCCTAGTGCCATCAGCTAA-3’ |
|  |  | *Potri.003G101500* | F: 5’-GGTTCAAGTAGCTATGGTATGGG-3’  R: 5’-CCATTCTGTCTCCAGGTCACTA-3’ |
|  | PAX (RIBOSOMAL PROTEIN S6 KINASE; associated with BRX, involved in sieve element differentiation) | *Potri.002G137700* | F: 5’-TCAAGGGCATATGTAGTGTCAG-3’  R: 5’-GATGTGCATTAAATCGACCCG-3’ |
| **AUTOPHAGY** | ATG8 (AUTOPHAGY-RELATED PROTEIN 8, molecular marker of autophagy) | *Potri.002G144600* | F: 5′-GCTGCGAGAATCAGGGAGAA-3′  R: 5′-CACAGTCAGGTCAGCAGGAA-3′ |
|  | ATG8 (AUTOPHAGY-RELATED PROTEIN 8, molecular marker of autophagy) | *Potri.014G060300* | F: 5′-GGAGGGCTGAAACTGCAAGA-3′  R: 5′-TCAGCCGGGACTAGGTACTTT-3′ |
|  | ATG8 (AUTOPHAGY-RELATED PROTEIN 8, molecular marker of autophagy) | *Potri.001G122700* | F: 5′-TGAGAAGAGGAGGGCAGAGG-3′  R: 5′-CAGTCAGGTCAGCTGGAACA-3′ |
|  | ATG8 (AUTOPHAGY-RELATED PROTEIN 8, molecular marker of autophagy) | *Potri.002G228800* | F: 5′-CCAATTTGTGTACGTGGTTCGG-3′  R: 5′-TGGCAGCAGTAGGTGGTAGAA-3′ |
|  | ATG8 (AUTOPHAGY-RELATED PROTEIN 8, molecular marker of autophagy) | *Potri.014G153800* | F: 5′-ACATCCTCTCGAAAGGAGGCAG-3′  R: 5′-GCAGCAGTTGGTGGTAGAATGT-3′ |
|  | ATG8 (AUTOPHAGY-RELATED PROTEIN 8, molecular marker of autophagy) | *Potri.004G013700* | F: 5′-ACTTTGGAGAGGAGGCAAGC-3′  R: 5′-GACCCACAGTCAAATCGGCT-3′ |
|  | ATG8 (AUTOPHAGY-RELATED PROTEIN 8, molecular marker of autophagy) | *Potri.011G004300* | F: 5′-CATGCTTTGGAGAGGAGGCA-3′  R: 5′-GGACATCACTCCTTCCAGCC-3′ |
|  | ATG8 (AUTOPHAGY-RELATED PROTEIN 8, molecular marker of autophagy) | *Potri.010G153400* | F: 5′-GGACCTGCCGGAAATGGAAA-3′  R: 5′-GGGCCTTTCCAGGAGTCAAC-3′ |
|  | ATG8 (AUTOPHAGY-RELATED PROTEIN 8, molecular marker of autophagy) | *Potri.008G099400* | F: 5′-CCGAGACATGACTATCGGGC-3′  R: 5′-AAATCCGTCATCGCCCTTGT-3′ |
| **SELECTIVE AUTOPHAGY** | BCS1 (CYTOCHROME BC1 SYNTHESIS; present in a homo-multimeric protein complex on the outer mitochondrial membrane and plays a role in cell death) | *Potri.T172000* | F: 5’-GTTCATCTCAAAACAAGTGCCA-3’  R: 5’-TCATGCCTCAGCGAGAAAA-3’ |
|  | NBR1 (NEXT TO BRCA1 GENE 1 protein; molecular marker of selective autophagy) | *Potri.012G085700* | F: 5’-GAAGATGGTGCAGGTTCAAGT-3’  R: 5’-CGCTGAAGTCCTGGTAACATT-3’ |
|  | ATG11 (AUTOPHAGY-RELATED PROTEIN 11, involved in mitophagy) | *Potri.006G180800* | F: 5’-GGTTTGGTAAATCAGACCAGG-3’  R: 5’-CGATATAACGCATAACCGCC-3’ |
|  |  | *Potri.018G103000* | F: 5’-GGTGTGGTAAATCAGCCCA-3’  R: 5’-TCAATACGCTGCATGACAGC-3’ |
|  | ATG5 (AUTOPHAGY-RELATED PROTEIN 5, involved in ribophagy) | *Potri.017G139700* | F: 5’-GCACATGTCTCAATCTGATCAAG-3’  R: 5’-CAACCATATCTGCCTCATCAGT-3’ |
|  | SAG20 (senescence associated gene 20-related protein) | *Potri.010G182200* | F: 5’-GGGTGTAATACAAGTCGTTCCA-3’  R: 5’-ACAGAGATATTTCGGGCGTTG-3’ |
|  | PUB4 (PLANT U-BOX 4; a functional ubiquitin-protein ligase which has been inferred to have roles in regulating root development and ROS-induced plastid degradation) | *Potri.005G144700* | F: 5’-AGCAGTCCCAGACATATAGC-3’  R: 5’-CTTTGGACACACAGTGAGC-3’ |
|  | IRE1B (ARABIDOPSIS THALIANA INOSITOL REQUIRING 1-1; involved in reticulophagy) | *Potri.002G007201* | F: 5’-GAATGTTGAATTGTGGAAGGCC-3’  R: 5’-TTCATGCAAATGGGCAAGCC-3’ |
|  | CHMP1B (CHARGED MULTIVESICULAR BODY PROTEIN/CHROMATIN MODIFYING PROTEIN1B; member of ESCRT-related proteins which mediates multivesicular body sorting of auxin carriers and required for plant development) | *Potri.001G194400* | F: 5’-GACTACCATTAACAAGTCTATGGC-3’  R: 5’-GTGGACAGCGAGGTAGAA-3’ |
|  |  | *Potri.003G045300* | F: 5’-CACCATTAACAAGTCCATGGG-3’  R: 5’-GGTAGACCCAGCCATAGAA-3’ |
|  | ATI1/ATI2 (ATG8-INTERACTING PROTEIN; partially associated with the ER during favourable growth conditions and becomes mainly associated with a spherical compartment that dynamically moves along the ER network, involved in autophagy of plastids or ER) | *Potri.014G086000* | F: 5’-CAAGGTTTATGAAGAAGATGAGGC-3’  R: 5’-CCTTCTTCTGGACCAAGTTCAA-3’ |
|  |  | *Potri.002G160700* | F: 5’-ATGGGGATGAAACTTCTCGG-3’  R: 5’-CCCTCTTCTAAACCAAGCTCAG-3’ |
|  | RPN10 (REGULATORY PARTICLE NON-ATPASE 10; regulatory particle non-ATPase subunit of the 26S proteasome with multiubiquitin-chain-binding capabilities, involved in proteaphagy) | *Potri.007G056200* | F: 5’-CTTACAATGGCTGGAAAAGGG-3’  R: 5’-ACCTGTATACCAGCAACCAAG-3’ |
|  |  | *Potri.009G133000* | F: 5’- CCAATGGATGAAGTGAATGCATTG-3’  R: 5-GAAGAGCTATAAATCCGGAACCC-3’ |
|  |  | *Potri.004G173700* | F: 5’-CCAATGGATGAAGTGAATGCTCTA-3’  R: 5’-AATGGAGATCTGAAGAGCCACT-3’ |
|  | ACD11 (ACCELERATED CELL DEATH 11; regulates mediators responsible for localized cellular death) | *Potri.006G051800* | F: 5’-GAAGATGAAAGCTCGGCAATAA-3’  R: 5’-CCAATCTATACCCAATTCTCTGGA-3’ |
| **ENUCLEATION** | NAC57 (phloem-specific NAC domain-containing transcription factor 57, regulating expression of genes required for enucleation) | *Potri.012G038100* | F: 5’-GCGAAGAAAGTAGGAGGTCAT-3’  R: 5’-TCTCAGGGATGATGTCTAAATCG-3’ |
|  |  | *Potri.015G030200* | F: 5’-CAAGGCCAATGCACAAGCA-3’  R: 5’-ACACCATGGCTCGTCTGTA-3’ |
|  | NAC75 (phloem-specific NAC domain-containing transcription factor 75, regulating expression of genes required for enucleation) | *Potri.018G068700* | F: 5’-CAGTTTGCAACTACTGCCTTT-3’  R: 5’-GTGGGTTTGCAATGGAAGAA-3’ |
|  |  | *Potri.006G152700* | F: 5’-GCGAAGAAAGTAGGAGGTCAT-3’  R: 5’-TCTCAGGGATGATGTCTAAATCG-3’ |
