# Supplementary figures and images for: Is autophagy always a death sentence? A case study of highly selective cytoplasmic degradation during phloemogenesis
Source: Ann Bot. 2024 Nov 5;135(4):681–96. doi: 10.1093/aob/mcae195 (PMC11904893; doi:10.1093/aob/mcae195)

PHLOEM DEVELOPMENT

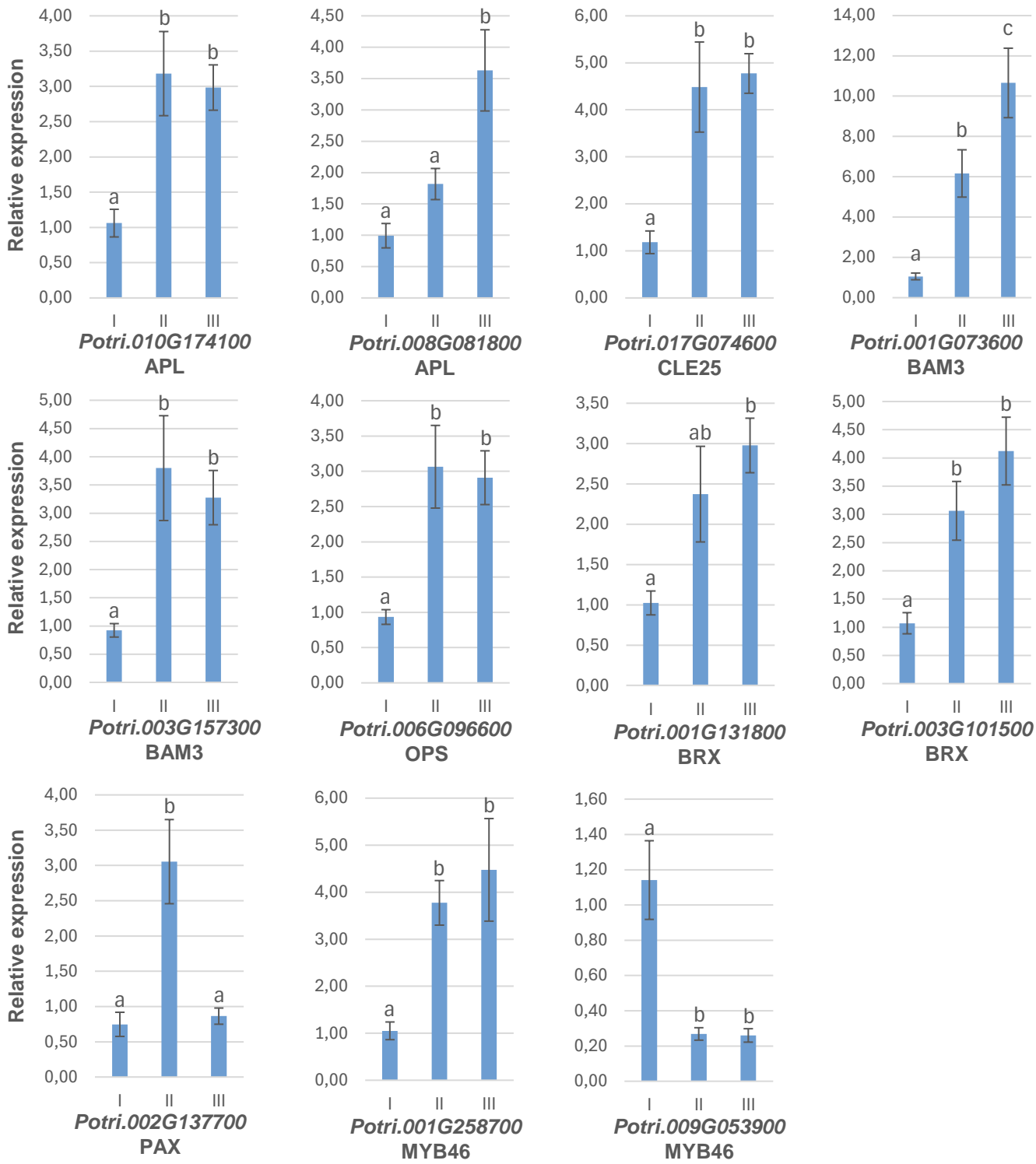

AUTOPHAGY

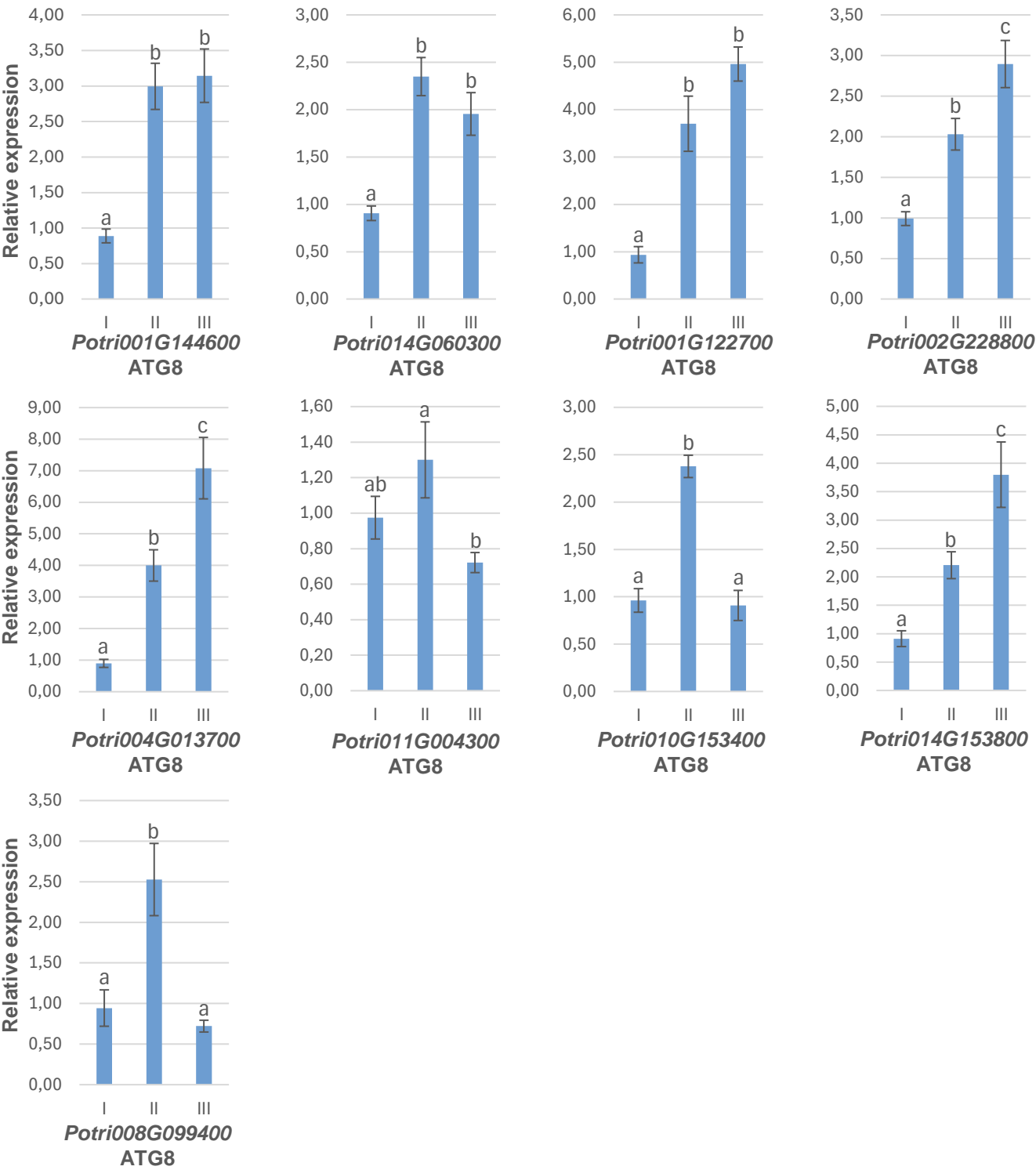

SELECTIVE AUTOPHAGY

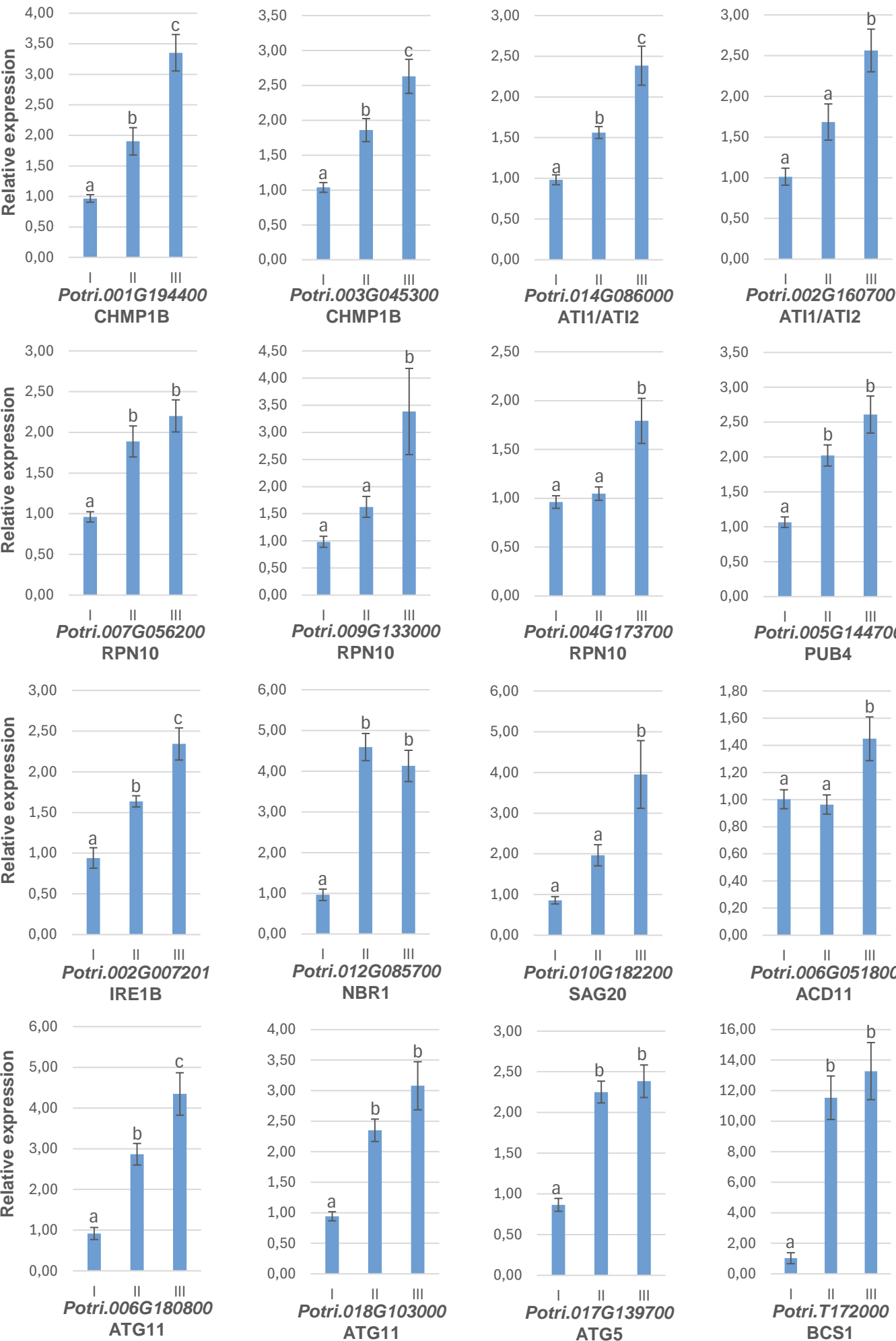

ENUCLEATION

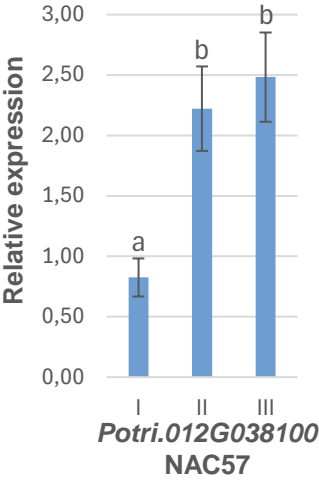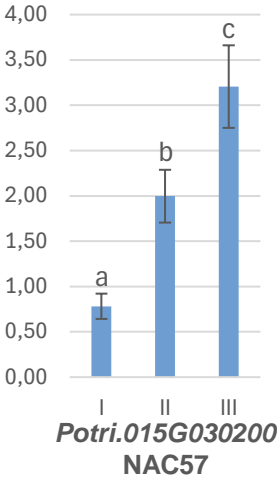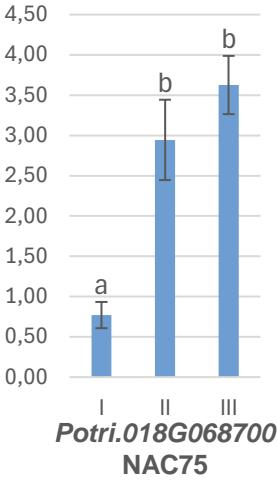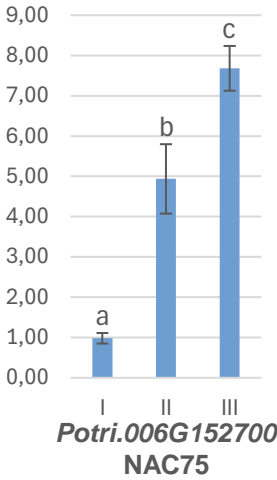

Supplement: mcae195_suppl_Supplementary_Figure_S1 [file mcae195_suppl_supplementary_figure_s1.pdf]

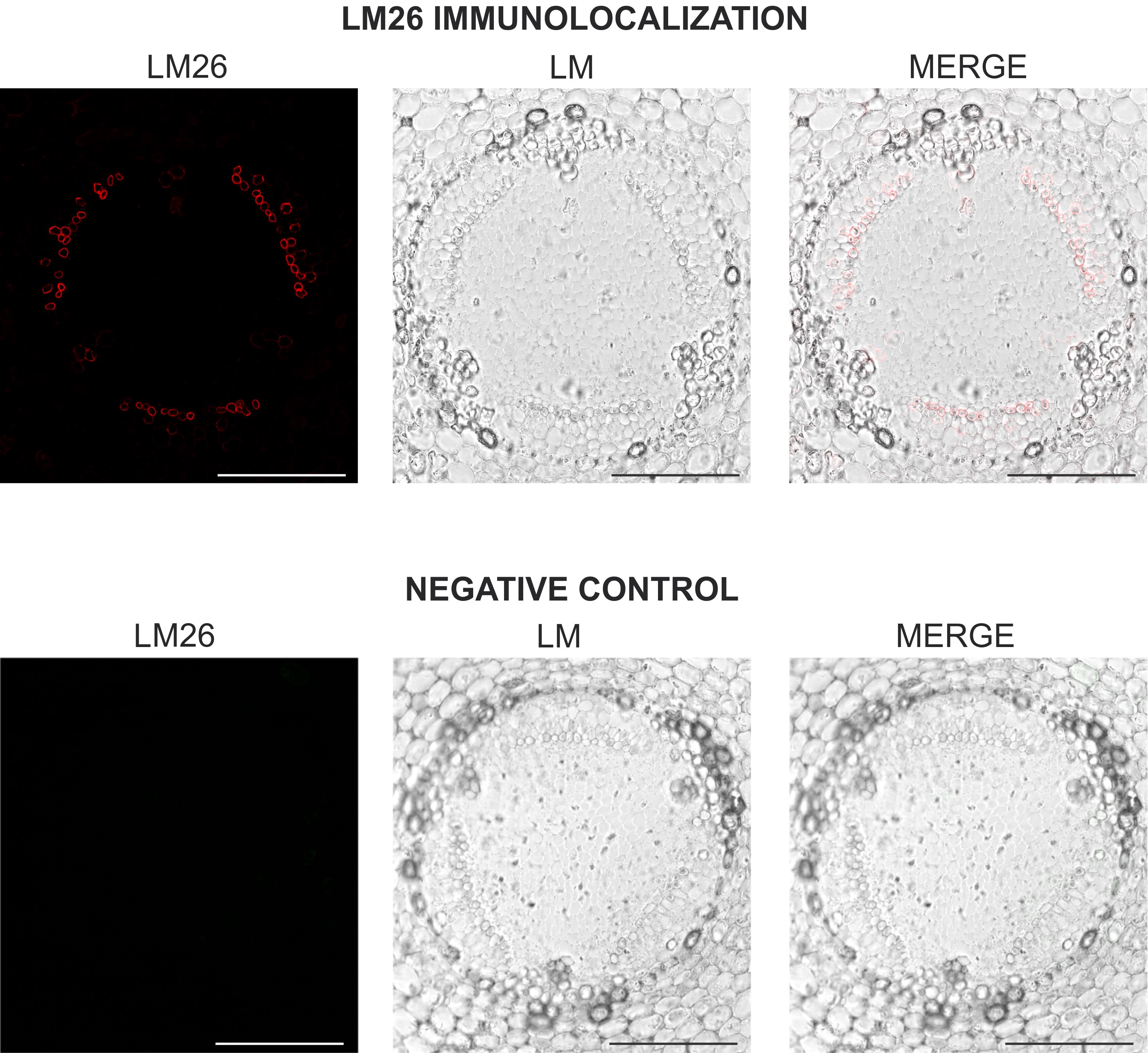

Supplement: mcae195_suppl_Supplementary_Figure_S2 [file mcae195_suppl_supplementary_figure_s2.jpeg]

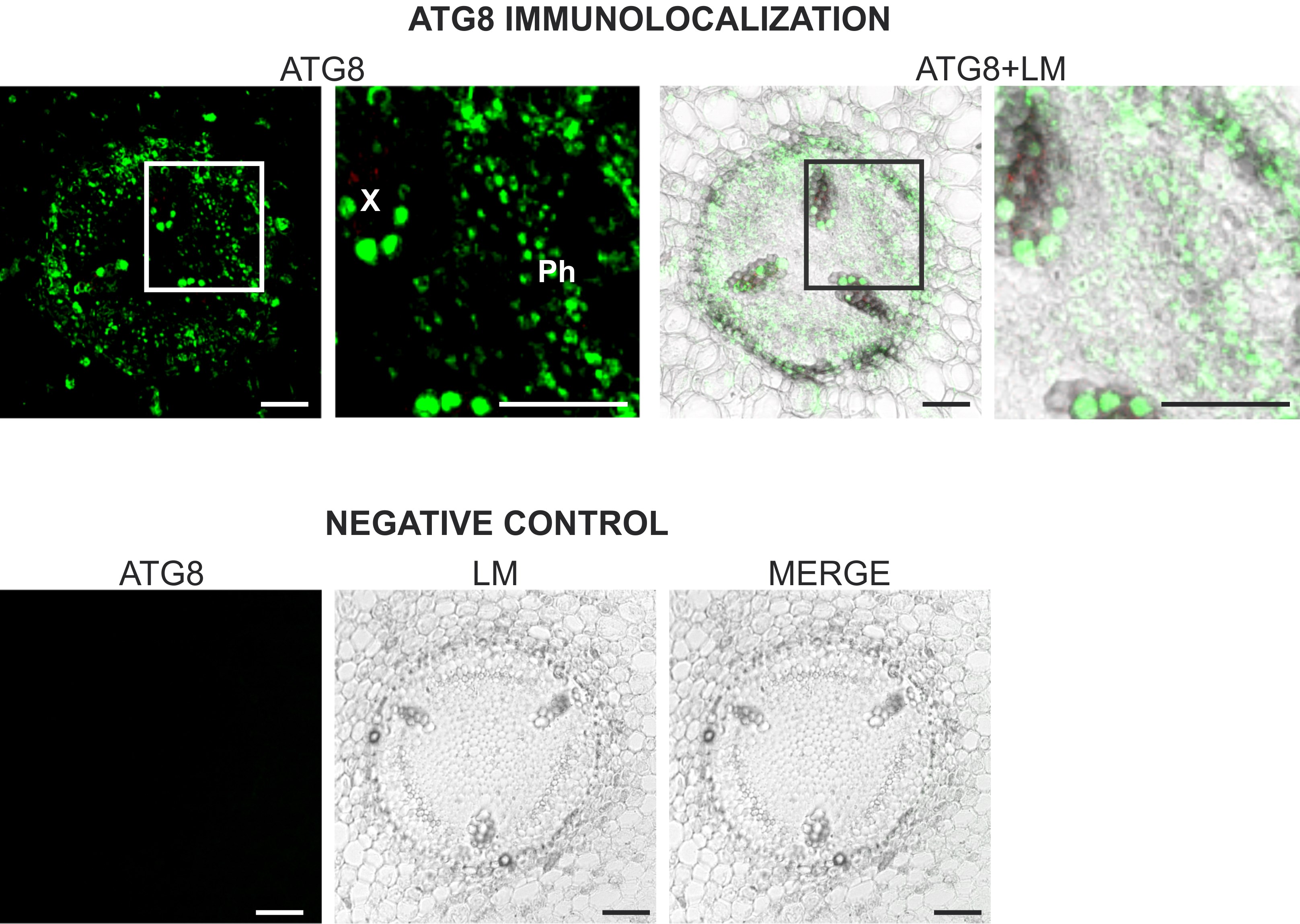

Supplement: mcae195_suppl_Supplementary_Figure_S3 [file mcae195_suppl_supplementary_figure_s3.jpeg]

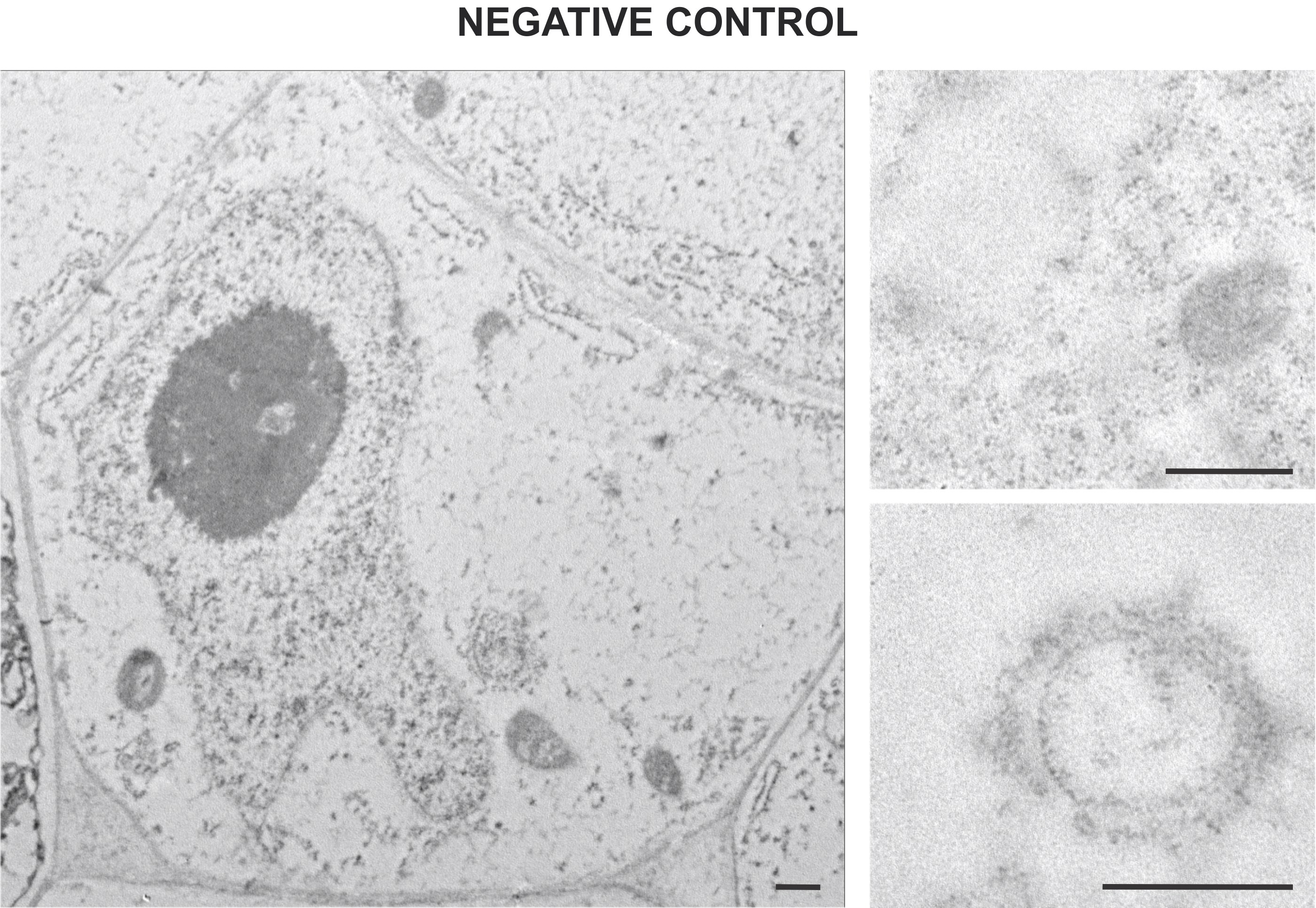

Supplement: mcae195_suppl_Supplementary_Figure_S4 [file mcae195_suppl_supplementary_figure_s4.jpeg]

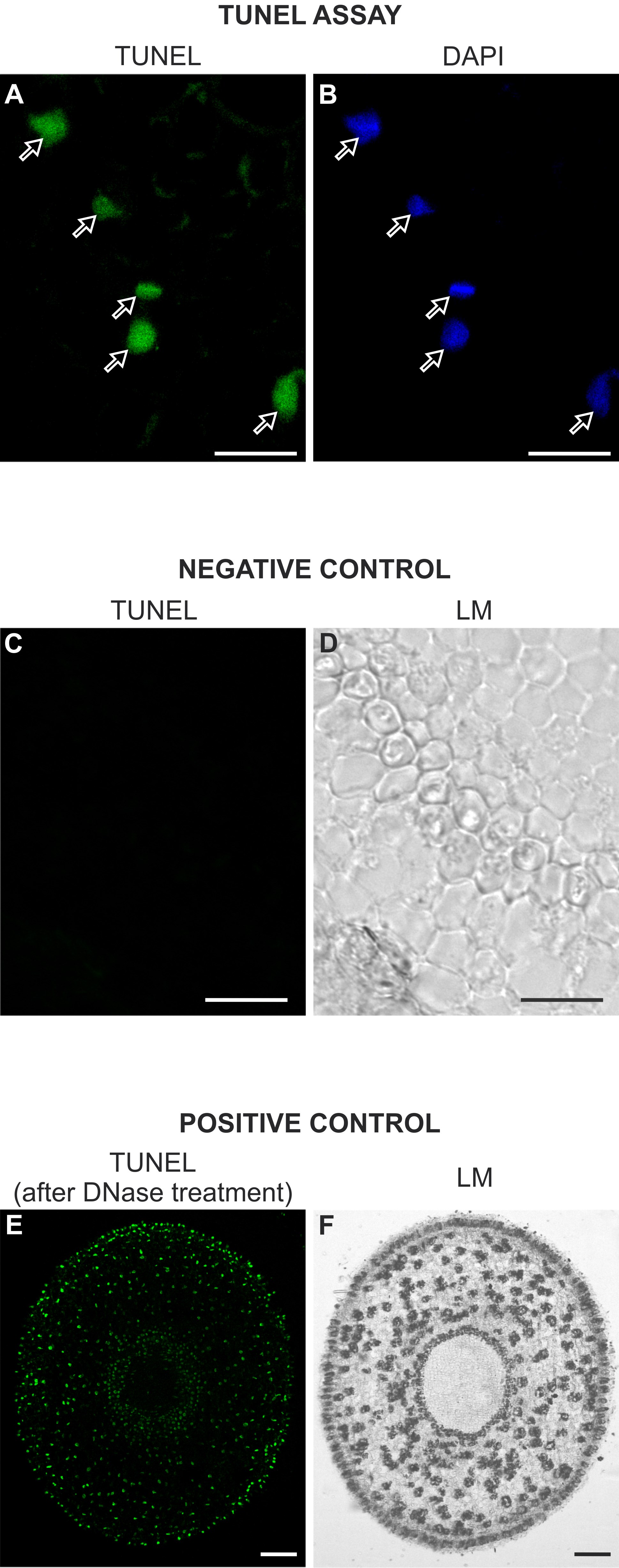

Supplement: mcae195_suppl_Supplementary_Figure_S5 [file mcae195_suppl_supplementary_figure_s5.jpeg]

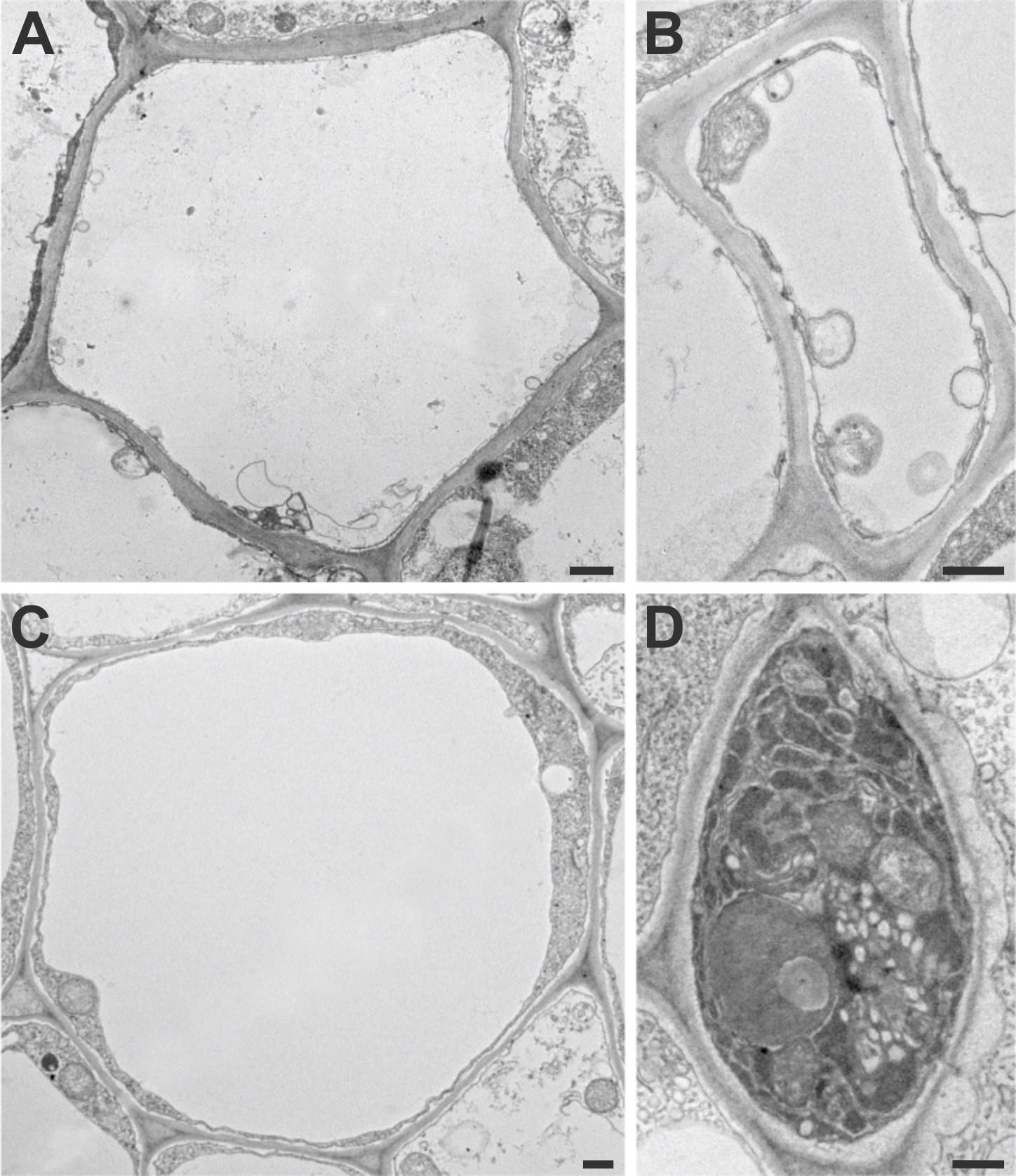

Supplement: mcae195_suppl_Supplementary_Figure_S6 [file mcae195_suppl_supplementary_figure_s6.jpeg]
